# Programmed ribosomal frameshifting during *PLEKHM2* mRNA decoding generates a constitutively active proteoform that supports myocardial function

**DOI:** 10.1126/sciadv.ady1742

**Published:** 2025-10-24

**Authors:** Gary Loughran, Raffaella De Pace, Ningyu Ding, Jianchao Zhang, Irwin Jungreis, Gionmattia Carancini, Jonathan M. Mudge, Ji Wang, Manolis Kellis, John F. Atkins, Pavel V. Baranov, Andrew E. Firth, Xiaowei Li, Juan S. Bonifacino, Yousuf A. Khan

**Affiliations:** ^1^ School of Biochemistry and Cell Biology, University College Cork, Cork, Ireland.; ^2^ Division of Neuroscience & Cellular Structure, Eunice Kennedy Shriver National Institute of Child Health and Human Development, National Institutes of Health, Bethesda, MD 20892, USA.; ^3^ Department of Cardiology, The First Affiliated Hospital of Zhengzhou University, Zhengzhou, Henan, Republic of China.; ^4^ Henan Key Laboratory of Hereditary Cardiovascular Diseases, Zhengzhou 450052, Republic of China.; ^5^ MIT Computer Science and Artificial Intelligence Laboratory, Cambridge, MA, USA.; ^6^ Broad Institute of MIT and Harvard, Cambridge, MA, USA.; ^7^ SFI CRT in Genomics Data Science, University of Galway, Galway, Ireland.; ^8^ European Molecular Biology Laboratory, European Bioinformatics Institute, Wellcome Genome Campus, Hinxton CB10 1SD Cambridge, UK.; ^9^ Department of Pathology, University of Cambridge, Cambridge, UK.; ^10^ Institute of Precision Medicine, The First Affiliated Hospital, Sun Yat-sen University, Guangzhou 510080, Republic of China.; ^11^ Department of Molecular and Cellular Physiology, Stanford University, Stanford, CA, USA.

## Abstract

Programmed ribosomal frameshifting is a process where a proportion of ribosomes change their reading frame on an mRNA. While frameshifting is commonly used by viruses, very few phylogenetically conserved examples are known in nuclear encoded genes. Here, we report a +1 frameshifting event during decoding of the human gene *PLEKHM2* that provides access to a second internally overlapping ORF. The new carboxyl-terminal domain of this frameshift protein forms an α helix, which relieves PLEKHM2 from autoinhibition and allows it to move to the tips of cells without activation by ARL8. Reintroducing both the canonically translated and frameshifted protein are necessary to restore normal contractile function of *PLEKHM2* knockout cardiomyocytes, demonstrating the necessity of frameshifting for normal cardiac activity.

## Teaser

PRF is a mechanism commonly used in viruses but rarely in eukaryotes; here, a conserved +1 PRF signal in PLEKHM2 is reported.

## INTRODUCTION

Ribosomes catalyze the synthesis of proteins, a tightly coordinated, regulated, and conserved process (*1*). During elongation, each codon of an mRNA is matched to its amino acid–specific transfer RNA (tRNA) in a sequential manner (*2*). A network of interactions between the ribosome, tRNAs, and other factors help to maintain translation fidelity and ensure that proteins are correctly synthesized (*3*). Thus, the rate of spontaneous frameshifting, where the ribosome shifts reading frame during elongation, is exceptionally low (*4*).

Programmed ribosomal frameshifting (PRF) occurs when a proportion of elongating ribosomes are induced to shift their reading frame by one or two nucleotides upon encountering specific signals in cis- or a trans-acting factor (*5*). Cis-acting PRF signals may include a slippery site where tRNAs can re-pair in an alternative reading frame, a 3′ RNA-element, or a nascent peptide that can interact with the ribosome’s peptide exit tunnel. Trans-acting stimulators of PRF include proteins (*6*, *7*) and small molecules such as polyamines (*8*). PRF typically results in ribosomes synthesizing a distinct polypeptide from a sequence 3′ of the PRF site, allowing for the generation of multiple polypeptides from one RNA. Many viruses—including severe acute respiratory syndrome coronavirus 2 (*9*), HIV-1 (*10*), and influenza (*11*)—require PRF for the expression of all their proteins from their genome. In vertebrates, there are few known examples of PRF required for the expression of nuclear genes. All vertebrate −1 PRF cases described to date are of viral origin (*12*, *13*) as reported cases of nonretrovirus-derived −1 PRF in vertebrate genes were shown to be artifacts (*14*, *15*). Antizyme mRNA requires +1 PRF to synthesize a single functional protein (*8*). While examples of translation from long overlapping reading frames in vertebrates are known (*16*, *17*), these result from leaky scanning or alternative splicing rather than PRF. An influenza virus-like +1 PRF site has been bioinformatically predicted in the vertebrate gene *ASXL1*, although this has not been experimentally validated (*18*). To date, there are no experimentally confirmed PRF signals in vertebrate cellular genes that provide ribosome access to internally overlapping ORFs, creating two proteins from one mRNA.

Here, we report the case of PRF in vertebrates, not of viral origin, to generate two proteins from one mRNA. We find a highly conserved +1 PRF site in the mRNA encoded by the gene *PLEKHM2* (also known as *SKIP*). PRF results in the synthesis of a transframe protein, PLEKHM2-frameshift (FS). This protein does not contain the autoinhibitory C-terminal domain of the canonically translated protein product, PLEKHM2-CT (*19*), and instead is predicted to contain an α-helical domain that we found to promote self-association. This results in a proteoform that does not require activation by ARL8 to promote kinesin-1–driven lysosome transport. We then infected *PLEKHM2* knockout human induced pluripotent stem cells that were differentiated into cardiomyocytes (hiPSC-CMs) with constructs either encoding for the PLEKHM2-CT, PLEKHM2-FS, or both proteoforms. We found that reintroducing only the PLEKHM2-CT proteoform did not significantly recover contractility, whereas reintroducing a combination of both the CT and FS proteoforms resulted in significant recovery.

## RESULTS

### PLEKHM2 expression involves a highly conserved +1 PRF

**Fig. 1.**
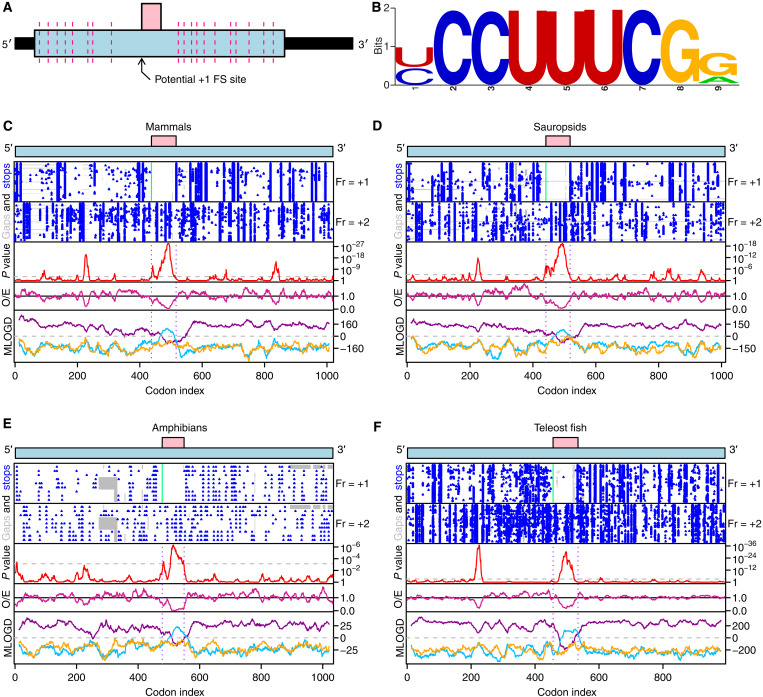
Synonymous site conservation and coding potential within the *PLEKHM2* dual coding region. (**A**) Schematic of the main *PLEKHM2* transcript. Pink-dashed lines indicate exon-exon junctions, and black bars represent untranslated regions (UTRs). The predicted PRF cassette is indicated with an arrow. The pink rectangle represents the +1 frame, conserved ORF. (**B**) Sequence logo of putative PRF cassette, generated from alignment of *PLEKHM2* mRNA sequences from mammals, sauropsids, amphibians, and teleost fish. (**C** to **F**) Synplot2 and MLOGD plots for mammals (C), sauropsids (D), amphibians (E), and teleost fish (F). Top schematic shows stop codons (blue) in aligned sequences in +1 frame. Next row shows stop codons in +2 frame. O/E plot shows ratio of observed to expected synonymous substitutions under a null model of neutral evolution at synonymous sites, and *P* value plot shows corresponding *P* value. Bottom row shows MLOGD coding potential plot for all three frames (purple, 0; blue, +1; yellow, +2).

We found a conserved overlapping ORF in one of the coding exons of *PLEKHM2* that could not be accessed by canonical translation of any RNA isoform (Fig. 1A). Toward the 5′ end of this alternative ORF is a potential +1 PRF sequence, UCC_UUU_CGG, almost identical to the influenza A virus UCC_UUU_CGU +1 PRF site, that is conserved in vertebrate *PLEKHM2* mRNAs (Fig. 1B). For the influenza A virus PRF, slippage is thought to occur when UUU is positioned in the ribosomal P-site and CGU is positioned in a presumably empty A site (*20*). Both UUU and UUC are decoded by the same tRNA isoacceptor whose anticodon, 3′-AAG-5′, has a higher affinity for UUC in the +1 frame than for the zero-frame UUU (*21*). Subjecting *PLEKHM2* transcript sequence alignments to both Synplot2 (*22*) and MLOGD (*23*) analysis, we found that in mammals, sauropsids, amphibians, and teleost fish, in the region of the *PLEKHM2* gene that is overlapped by the *FS* ORF, there is statistically significantly enhanced synonymous site conservation and a positive coding signature specifically in the +1 reading frame (Fig. 1, C to F).

**Fig. 2.**
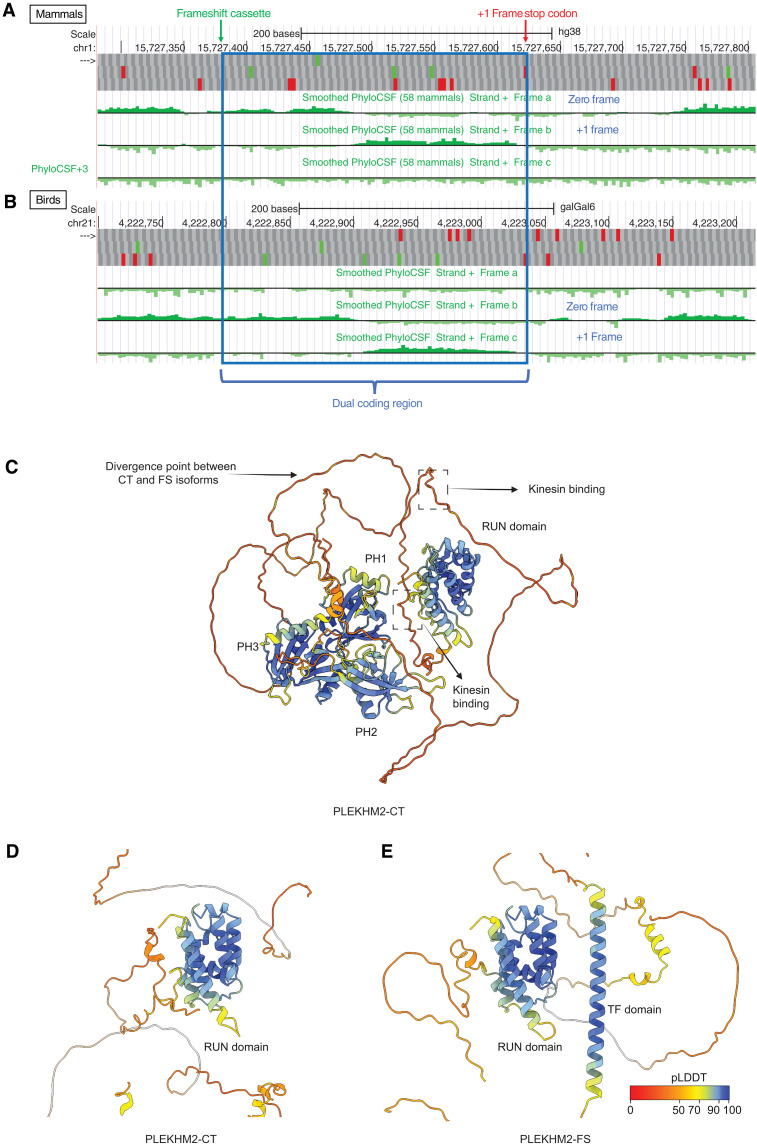
PhyloCSF and AlphaFold2 predictions on PLEKHM2. (**A** and **B**) UCSC Genome Browser tracks showing evolutionary signature of protein coding potential in each of three reading frames as measured by PhyloCSF in mammals (A) and birds (B). Part way through the dual coding region (blue) between the PRF cassette (green arrow) and the +1 frame stop codon (red arrow), the PhyloCSF signal changes to the +1 frame, indicating greater amino acid conservation in the +1 frame and then resumes in the zero frame downstream of the +1 frame stop codon. (**C**) ColabFold predictions of PLEKHM2-CT. PH, pleckstrin homology. (**D**) Zoomed-in view of RUN domain of PLEKHM2-CT. (**E**) ColabFold prediction of PLEKHM2-FS. pLDDT coloring scheme is shown in the bottom right and applies to (C) to (E).

PhyloCSF (*24*), in addition to MLOGD and Synplot2 analysis, also independently indicates a conserved change of reading frame in exon 9 of *PLEKHM2*, followed by coding resumption in the original reading frame downstream of the *FS* stop codon, in both mammals and birds (Fig. 2, A and B) (*25*). A whole-genome alignment of 100 vertebrate species shows that (i) both the CC_UUU_C 6-mer in the key portion of the potential PRF cassette and the *FS* stop codon are present in all aligned species; (ii) there is a preponderance of synonymous substitutions relative to the +1 frame in the latter portion of the potential dual coding region, indicating purifying selection on the amino-acid sequence translated from that frame; and (iii) there are no stop codons in the +1 frame within the potential dual coding region despite many stop codons in the flanking sequences on both sides of the aligned sequences (fig. S1, A to D). Thus, three different methods for identifying overlapping reading frames and/or elements (MLOGD, Synplot2, and PhyloCSF) all detect this *FS* ORF in vertebrates.

Using ColabFold (*26*) and assuming PRF on UCC_UUU_CGG, we input the sequence of the CT and predicted FS protein. ColabFold reliably predicted the N-terminal portion of the CT protein (Fig. 2, C and D), and we generated custom multiple sequence alignments for PLEKHM2-FS across all vertebrates, injected them into the model and were able to predict an additional high confidence α-helical domain in the transframe portion of the FS protein (Fig. 2E).

### Experimental evidence of +1 PRF in PLEKHM2

**Fig. 3.**
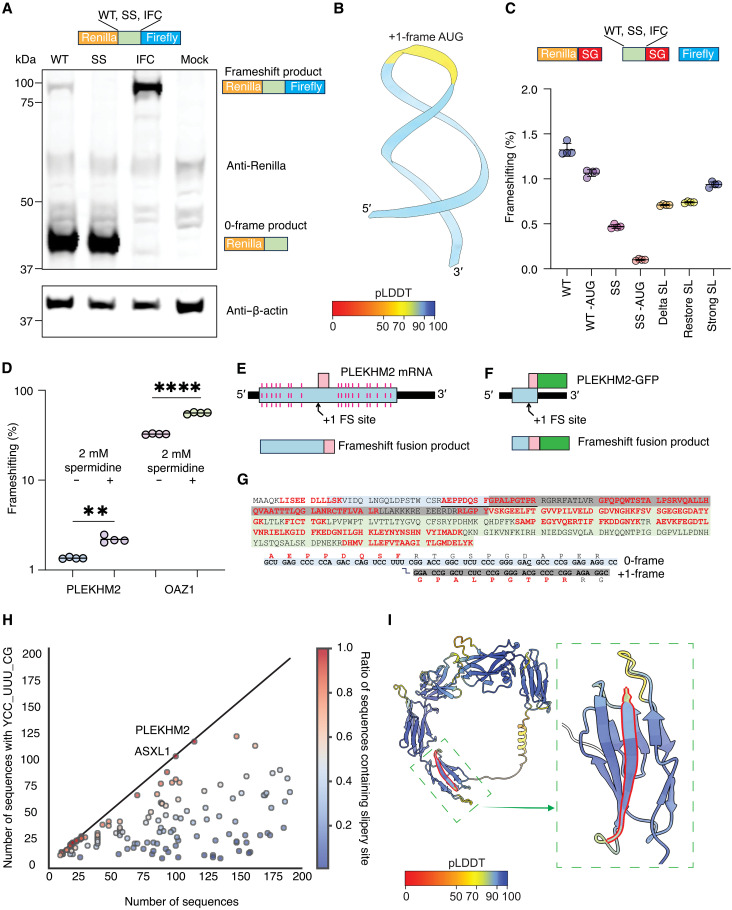
PRF on *PLEKHM2* mRNA. (**A**) Representative immunoblot of whole-cell lysates prepared from HEK293T cells transfected with *PLEKHM2* dual luciferase constructs. WT, wild-type (UCC_UUU_CGG); SS, frameshift mutant (agC_UUc_aGa); IFC, in-frame control (agC_UUc_Ga) with both reporters in the zero-reading frame; MOCK, no plasmid transfected. (**B**) AlphaFold3 prediction of downstream RNA structure to the slippery site. RNA backbone is shown in a cartoon representation with nucleotides colored by pLDDT. The three nucleotides that show low pLDDT confidence (yellow) are the +1 frame AUG codon. (**C**) PRF efficiencies (%) of *PLEKHM2* determined by dual luciferase assays. WT, wild-type (UCC_UUU _CGG); SS, frameshift mutant (agC_UUc_aGa). Change of +1 frame AUG to ACG labeled as “-AUG”. SG, StopGo sequence. Delta SL, restore SL, and strong SL changes are depicted in fig. S2B. *n* = 4. (**D**) PRF efficiencies for *PLEKHM2* and *OAZ1* PRF cassettes upon addition of 2 mM spermidine (SPD). *n* = 4. (**E**) Schematic depicting how mRNA of *PLEKHM2* corresponds to frameshifted fusion product. (**F**) Schematic of MS construct. (**G**) MS results, with identified peptides highlighted in red font and junction peptide underlines. Junction peptide coverage shown below, with line depicting the transition from 0 to +1 frame, confirming the site of PRF. (**H**) Plot depicting the degree to which human genes containing +1 PRF motif YCC_UUU_CG are conserved across sequence alignments. Points are colored by the fraction of aligned sequences of a gene containing the +1 PRF motif. (**I**) Prediction of putative frameshift generated peptide from NUP210L. The red highlight shows the C-terminal frameshift peptide generated.

Possible PRF into the +1 reading frame was tested experimentally by inserting the putative frameshift site and flanking sequence from *PLEKHM2* between *Renilla* and firefly luciferase CDS (coding sequence)s such that a +1 PRF event would give rise to a ~100-kDa fusion product of the two reporter polypeptides in human embryonic kidney (HEK) 293T cells. This demonstrated PRF in a wild-type (WT) construct, but not upon mutation of the putative PRF cassette from UCC_UUU_CGG to agC_UUc_aGa (Fig. 3A).

To measure the PRF efficiency of *PLEKHM2*, we used a dual luciferase reporter system that we previously described (Fig. 3, B and C) (*27*). This arrangement avoids potential artifacts that can arise when the test sequence fusion alters the individual reporter activities or stabilities (*14*, *15*, *27*). Furthermore, we changed a +1 frame AUG within the *PLEKHM2* cassette to minimize the possibility of firefly luciferase translation due to initiation on potential low abundance mRNAs that may be generated by cryptic splicing or cryptic promoters. Dual luciferase assays in HEK293T cells indicated a substantial PRF efficiency of ~1.3% for WT and ~1.1% for WT minus AUG (Fig. 3C and fig. S2, A and B). For reference, HIV-1 has been shown to frameshift at an efficiency of ~2% (*14*) and influenza at ~1% (*11*).

We noticed a stem-loop 3′ of the *PLEKHM2* shift site that appears to display sequence conservation (fig. S3A) and was predicted confidently by AlphaFold3 (Fig. 3B) (*28*). To test the possible stimulatory role of this putative stem-loop structure, we introduced mutations to disrupt (delta SL), restore (restore SL), or strengthen (strong SL) the stem-duplex (fig. S3B). Disrupting the putative stem-loop structure decreased *PLEKHM2* PRF efficiency to ~0.75%. However, WT levels of PRF were not restored by changes that were predicted to restore stem-loop formation or strengthen the stem-loop (Fig. 3C and fig. S2, A and B), indicating that similar to influenza, *PLEKHM2* only requires its slippery site to induce +1 PRF.

We tested whether +1 PRF in the *PLEKHM2* gene may also be stimulated by polyamines because polyamines have been proposed to stimulate +1 PRF in a sequence-independent manner (*29*, *30*). Although baseline *OAZ1* PRF (32.5%) is much higher than *PLEKHM2* (~1%), we observed a similar 1.5-fold increase in PRF efficiency for both *OAZ1* and *PLEKHM2* when spermidine was added (Fig. 3D and fig. S2, C and D). This indicates that *PLEKHM2* PRF efficiency can be regulated and operates in a manner similar to other +1 PRF motifs. To assess whether *PLEKHM2* PRF may be more efficient in other cells or tissues, we compared ribosome read densities extracted from publicly available ribosome profiling data, upstream and downstream of the PRF cassette in the *PLEKHM2* mRNA. Although subtle differences in read densities were observed among different tissues, the baseline rate of PRF is too low to determine whether these differences are significant (fig. S4).

To determine the precise site and direction of PRF, we generated a construct in which a CDS encoding green fluorescent protein (GFP) was fused in-frame to the 3′ end of the transframe CDS (Fig. 3, E and F). An additional construct (PLEKHM2-K-GFP) introducing a lysine codon close to the slip site was used to increase the chances of identifying the junction tryptic peptide via mass spectrometry (MS) (fig. S2E). Constructs were expressed in HEK293T cells, and transframe fusions were affinity-purified from cell lysates and resolved by SDS–polyacrylamide gel electrophoresis (SDS-PAGE). Both constructs directed synthesis of a specific protein migrating at the predicted molecular weight for PLEKHM2-FS-GFP (~34 kDa). Tryptic peptides covering 59% of the PLEKHM2-FS-GFP construct and 85% of the PLEKHM2-K-FS-GFP construct were identified via liquid chromatography tandem MS (LC-MS/MS), including peptides encoded both upstream and downstream of the shift site (Fig. 3G and fig. S2E). Three peptides spanning the shift site itself were identified. These peptides—AEPPDQSFGPALPGTPR (from PLEKHM2-FS-GFP), QSFGPALPGTPR, and AEPPKQSFGPALPGTPR with a missed tryptic cleavage (from PLEKHM2-K-FS-GFP)—define the shift site (UCC_UUU_CGG) and direction (+1) of PRF. In addition, a portion of the *FS* ORF was identified in a previous study that used ribosome profiling and MS of human cell lines (*31*).

### Widespread occurrence of +1 PRF motif in vertebrate mRNAs

Given that the YCC_UUU_CG +1 PRF motif stimulates substantial levels of frameshifting in *PLEKHM2* (Fig. 3C) and influenza A virus (*11*, *20*) without the necessity of any additional factors, we investigated the presence of this motif in other vertebrate mRNAs. Starting with sequence alignments of 17,922 human CDSs with their homologs selected at a ≥65% amino acid identity threshold (Materials and Methods), we found 4128 occurrences of YCC_UUU_CG in all queried and aligned CDSs (mostly vertebrate, a few chordate) with variable levels of conservation, with some being present in nearly every species to those present in only a few (Fig. 3H). Given this, there is potential that phylogenetically restricted [narrowly conserved and/or weakly conserved noncanonical reading frames in mammals with clear and critical cellular roles have been shown before (*32*)] frameshifting occurs at these narrowly conserved CDSs as opposed to the exceptionally conserved case presented here for *PLEKHM2*. For example, NUP210L only had eight total sequences in our alignments of organisms, five of which contained the slippery sequence. Despite its limited conservation, human NUP210L’s frameshift product encodes a protein product that is confidently predicted as a β strand completing the domain it is present in (Fig. 3I). It has been previously shown that many key physiochemical properties of proteins remain the same in alternative frames and that this feature could allow for the creation of new proteins if these frames were accessed (*33*). Thus, it stands to reason the emergence of a +1 PRF motif would allow for the creation and selection of de novo proteins. However, the only way to truly confirm if a de novo +1 PRF-generated peptide is relevant is through extensive functional analysis.

### The PLEKHM2-FS proteoform is constitutively active

**Fig. 4.**
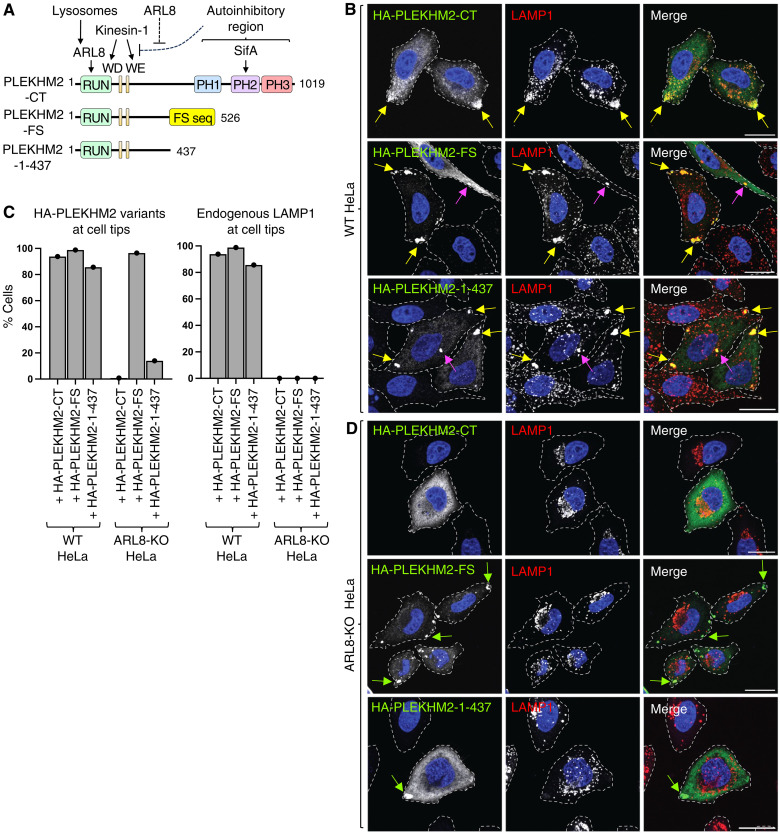
The PLEKHM2-FS proteoform is constitutively active in driving lysosome movement toward the cell periphery. (**A**) Schematic representation of PLEKHM2 variants. The different domains/motifs are indicated by colored boxes: RPIP8, UNC-14, and NESCA (RUN); Trp-Asp (WD); Trp-Glu (WE); pleckstrin homology (PH). Arrows and dashed lines indicate positive and negative interactions, respectively, with binding partners. Amino-acid numbers are also indicated. (**B** to **D**) WT HeLa cells were transiently transfected with the indicated HA-tagged PLEKHM2 constructs for 48 hours and analyzed by confocal immunofluorescence microscopy for the HA epitope (green) and endogenous LAMP1 (lysosomes, red). Nuclei were labeled with 4′,6-diamidino-2-phenylindole (DAPI) (blue). Cell edges were outlined by staining of actin with Alexa Fluor 647–conjugated phalloidin (not shown) and indicated by dashed lines. Arrows in (B) and (D) indicate clusters of PLEKHM2 together with lysosomes (yellow arrows) or without lysosomes (green arrows) at cell tips or near the cell center (magenta arrows). Scale bars, 20 μm. (C) Quantification of the percentage of WT (B) and ARL8-KO cells (D) displaying accumulation of PLEKHM2 variants or LAMP1 at cell tips. More than 150 cells per sample were scored. (D) ARL8-KO HeLa cells were transfected and analyzed as described for (B). Scale bars, 20 μm.

Next, we investigated the activity of the PLEKHM2-FS proteoform. PLEKHM2-CT (*34*) comprises a RUN domain that interacts with the lysosome-associated small guanosine triphosphatase ARL8 (*19*), WD-WE motifs that interact with kinesin-1 (*35*), a largely disordered region, and three autoinhibitory PH domains (Fig. 4A) (*19*). ARL8 both recruits PLEKHM2-CT to lysosomes and relieves PLEKHM2-CT autoinhibition (*34*). These properties enable PLEKHM2-CT to function as a regulated adaptor for ARL8-dependent coupling of lysosomes to kinesin-1 and consequent transport toward the plus end of microtubules (*19*, *35*).

The PRF that generates PLEKHM2-FS preserves the RUN domain and WD-WE motifs but parts of the disordered region and the three PH domains are absent (Fig. 4A). As previously shown (*34*, *35*), the overexpression of hemagglutinin (HA)–tagged PLEKHM2-CT (HA-PLEKHM2-CT) in WT HeLa cells (fig. S5A) results in the redistribution of a large fraction of lysosomes immunolabeled for the lysosomal markers LAMP1 (Fig. 4, B and C, and fig. S6A) and LAMTOR4 (fig. S5B) to cell tips. The overexpression of HA-PLEKHM2-FS or a truncated HA-PLEKHM2 1-437 lacking the three PH domains and the FS sequence (Fig. 4A and fig. S5A) similarly redistributed lysosomes to cell tips (Fig. 4, B and C, and fig. S5B and S6A). These observations demonstrated that PLEKHM2-FS is at least as active as PLEKHM2-CT and that this activity does not depend on the PLEKHM2-FS sequence.

Knockout (KO) of both the ARL8A and ARL8B paralogs in HeLa cells (ARL8 KO) prevented the association of HA-PLEKHM2-CT with lysosomes and kinesin-1 thereby impeding the overexpression-induced redistribution of both lysosomes and PLEKHM2 to cell tips (Fig. 4, C and D, and fig. S5A). Although overexpressed HA-PLEKHM2-FS failed to redistribute lysosomes, it could itself relocate to the tips of ARL8-KO cells (Fig. 4, C and D, and fig. S5C). This latter phenotype differs from that observed for HA-PLEKHM2-CT and is likely attributable to the absence of the autoinhibitory PH domains in PLEKHM2-FS. These observations indicated that PLEKHM2-FS is constitutively active, requiring no activation by ARL8. However, while PLEKHM2-FS may not require ARL8 for activation, it still requires ARL8 to associate with lysosomes and transport them.

In ARL8-KO cells, the truncated HA-PLEKHM2-1-437 exhibited a more cytosolic distribution (Fig. 4D and figs. S5C and S7, A and B) and substantially reduced movement to cell tips compared to PLEKHM2-FS (Fig. 4, C and D, and figs. S5C, S6B, and S7, A and B). These observations suggest that the FS sequence does not just replace an autoinhibitory sequence but also enhances the interaction with kinesin-1. Coimmunoprecipitation of HA- and myc-tagged constructs showed that PLEKHM2-FS interacted with itself much more than with PLEKHM2-CT or PLEKHM2-1-437 (fig. S5D).

We were then curious how these different PLEKHM2 isoforms differed in their affinity to kinesin. We transfected HEK293T with our constructs (Fig. 4A) and then performed immunoprecipitation with an HA antibody. We then took these samples and immunoblotted for KIF5B (kinesin-1 heavy chain). We found that the HA-PLEKHM2-FS isoform pulled down significantly more KIF5B than the HA-PLEKHM2-WT isoform (fig. S8, A and B).

These data demonstrate that the constitutively active PLEKHM2-FS proteoform likely plays a role in remodeling the lysosome distribution in the cell via its enhanced interaction with kinesin-1, which is potentially mediated by the FS sequence’s propensity to self-associate.

### The PLEKHM2-FS proteoform contributes to CM contraction activity

Proper lysosomal function and positioning is critical for autophagy, a conserved process for bulk degradation and recycling of cellular components (*36*) and plays an essential role in the heart (*37*, *38*). A two-base deletion mutation in PLEKHM2 was identified in patients suffering from dilated cardiomyopathy (DCM) and left ventricular noncompaction (LVNC) (*39*). In fibroblasts derived from these patients, normal lysosomal distribution was perturbed, leading to impaired autophagy flux and thereby providing a mechanism for PLEKHM2’s role in myocardial contraction. Furthermore, the disruption of PLEKHM2 splicing has also been shown to lead to DCM and subsequent death (*40*). This led us to investigate the potential role of the PLEKHM2 proteoforms in cardiac function.

**Fig. 5.**
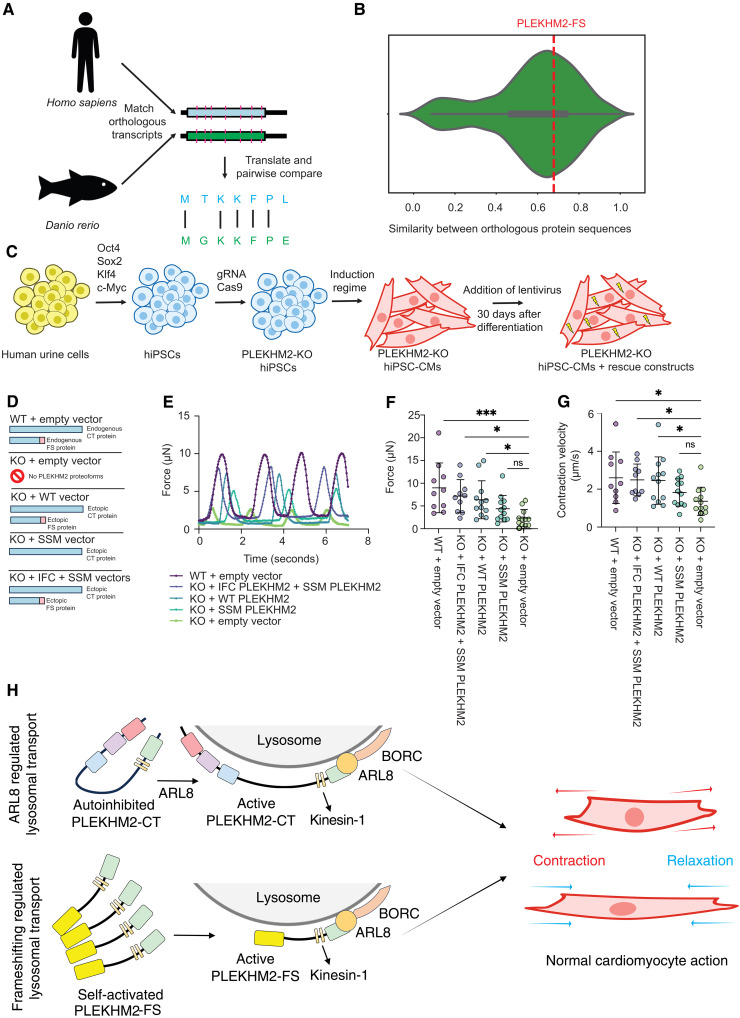
The PLEKHM2-FS proteoform contributes to cardiac function. (**A**) Schematic representation of the generation of a set of conserved proteins between *H. sapiens* and *Danio rerio* and computing pairwise orthology. (**B**) Distribution of the similarity of conserved protein sequences in vertebrates, with a red line representing the degree of PLEKHM2-FS proteoform conservation. (**C**) Schematic representation of the process of establishing hiPSC-CMs. (**D**) Schematic representation of the different constructs used to infect hiPSC-CMs, illustrating which proteoforms (CT and FS) are expressed in the cell by each construct combination. (**E**) Example of a single force trace experiment. (**F** and **G**) Quantification of force and contraction velocity in WT hiPSC-CMs and PLEKHM2-KO hiPSC-CM expressing different constructs analyzed with a one-way analysis of variance (ANOVA) [not significant (ns): *P* value > 0.05, *P value < 0.05, **P value < 0.01, and ***P value < 0.001]. Each dot represents a set of measurements from an individual cell (*n* = 10 to 12). (**H**) Schematic representation of the BORC-ARL8–dependent activation and recruitment of PLEKHM2 to a lysosome (top), and the recruitment of the constitutive, ARL8-independent activity but ARL8-dependent recruitment of PLEKHM2-FS to a lysosome (bottom), and how they support cardiomyocyte function.

Given that the chambered heart system arose evolutionarily in vertebrates (*41*), we wanted to see how conserved the PLEKHM2-FS protein sequence was relative to other key vertebrate proteins. By determining the set of all conserved proteins between *Homo sapiens* and *Danio rerio*, we were then able to pairwise compare every single protein sequence and determine a conservation distribution (Fig. 5, A and B). PLEKHM2-FS, also conserved in all vertebrates, was more conserved than the majority of orthologous proteins (Fig. 5B), suggestive of its essential role in vertebrate biology.

To further investigate PLEKHM2-FS’s physiological role, we used CRISPR-generated PLEKHM2-KO hiPSCs (Fig. 5C and Materials and Methods) (*42*). These cells were then differentiated into CMs (hiPSC-CMs) (*43*). These hiPSC-CMs were next infected with lentivirus containing either an empty vector, the WT PLEKHM2 cDNA, PLEKHM2 cDNA with a silent slippery site mutant (SSM), or with two vectors: SSM PLEKHM2 and PLEKHM2 cDNA with an in-frame control mutation. A control cell line where PLEKHM2 was not knocked out was also established and infected with an empty vector (Fig. 5D). As expected, the PLEKHM2-KO hiPSC-CMs showed ablated myocardial contractility (Fig. 5, E to G, and fig. S9, A and B), elevated levels of heart failure biomarkers NPPA and NPPB (atrial natriuretic peptide and B-type natriuretic peptide, respectively) (*44*) (fig. S9, B and C), abnormal calcium handling (fig. S9, D to H), and an accumulation of autophagy receptor p62/SQSTM1 (fig. S9, I and J), while most conditions reintroducing PLEKHM2 proteoforms to these PLEKHM2-KO hiPSC-CMs showed various degrees of recovery at or toward WT hiPSC-CMs.

Notably, however, PLEKHM2-KO hiPSC-CMs reinfected with the PLEKHM2 SSM vector, diminishing its ability to productively frameshift and therefore only producing the PLEKHM2-CT proteoform (Fig. 3B), were not able to significantly recover any aspect of myocardial contraction including force (Fig. 5, E and F), contraction velocity (Fig. 5G), average contraction displacement (fig. S9A), or relaxation velocity (fig. S9B). Upon reintroduction of the PLEKHM2-CT proteoform along with the -FS proteoform, significant myocardial contraction was indeed restored (Fig. 5, E to G, and fig. S9, A and B), demonstrating the critical role that both the PLEKHM2-CT and PLEKHM2-FS proteoforms play in contraction.

## DISCUSSION

Based on these data, we conclude that PLEKHM2-FS is a constitutively active variant of PLEKHM2 that does not require ARL8-dependent activation for kinesin-1–dependent movement of lysosomes toward the cell periphery. This proteoform, generated by +1 PRF with a slippery site similar to the one identified in influenza, is necessary along with the PLEKHM2-CT proteoform to promote myocardial contraction (Fig. 5H).

Although the conservation of PLEKHM2-FS in vertebrates coincides with the development of the modern, chambered heart, we do not rule out that PLEKHM2-FS may be involved in other physiological processes dependent on lysosomal distribution and therefore autophagy in the nervous system (*45*), immunity (*46*), and cancer (*47*).

How the presence of this constitutively active form of PLEKHM2 specifically affects the dynamics of kinesin-1–driven lysosomal distribution within cells remains an open question. PLEKHM2-FS could provide a basal level of lysosome transport, while PLEKHM2 would enable another level of regulated lysosome transport in response to stimuli such as starvation (*48*) or growth factor receptor activation (*49*). Furthermore, PLEKHM2-FS may not just be constitutively activated but could also play a role in activating the nonframeshift proteoform.

The discovery of the first PRF signal in vertebrate cellular genes that provides ribosome access to internally overlapping ORFs thus marks a major milestone moving this phenomena beyond viruses and sets the stage for further searches in vertebrate genomes. At a minimum, we know that the identified mRNAs in this study containing this confirmed +1 PRF slippery site motif very likely +1 frameshift efficiently, but it remains to be determined whether the resulting trans-frame proteins have biological functions and what those may be. As additional PRF elements with different slippery site motifs are identified, we expect the number of identified PRF elements in cellular genomes to continue to increase.

## MATERIALS AND METHODS

### Bioinformatic analysis

Reference sequences representing mammals, bird, amphibians, and fish were used to generate sequences for each of these clades, in a manner previously reported (*16*). For each clade, the sequences were translated to amino acids, aligned using MUSCLE (*50*), and the amino acid alignments were used to guide codon-respecting nucleotide sequence alignments using EMBOSS tranalign (*51*). For the MLOGD and synplot2 analyses, the alignments were mapped to the coordinates of a specific reference sequence by removing alignment columns that contained a gap character in the reference sequence. The reference sequences used were the coding regions of National Center for Biotechnology Information (NCBI) accession numbers NM_015164.4 (*H. sapiens*), XM_417616.8 (*Gallus gallus*), XM_041569881.1 (*Xenopus laevis*), and NM_001130783.1 (*D. rerio*) for the mammal, sauropsid, amphibian, and teleost fish alignments, respectively. These alignments were analyzed with synplot2 using a 15-codon window size and MLOGD using a 25-codon window size. The sequence of the human, PLEKHM2-FS protein sequence is MEPGEVKDRILENISLSVKKLQSYFAACEDEIPAIRNHDKVLQRLCEHLDHALLYGLQDLSSGYWVLVVHFTRREAIKQIEVLQHVATNLGRSRAWLYLALNENSLESYLRLFQENLGLLHKYYVKNALVCSHDHLTLFLTLVSGLEFIRFELDLDAPYLDLAPYMPDYYKPQYLLDFEDRLPSSVHGSDSLSLNSFNSVTSTNLEWDDSAIAPSSEDYDFGDVFPAVPSVPSTDWEDGDLTDTVSGPRSTASDLTSSKASTRSPTQRQNPFNEEPAETVSSSDTTPVHTTSQEKEEAQALDPPDACTELEVIRVTKKKKIGKKKKSRSDEEASPLHPACSQKKCAKQGDGDSRNGSPSLGRDSPDTMLASPQEEGEGPSSTTESSERSEPGLLIPEMKDTSMERLGQPLSKVIDQLNGQLDPSTWCSRAEPPDQSFGPALPGMPRRGRRFATLVRGFQPQWTSTALPSRVQALLHQVAATMTLQGLANRCMFLVALRLLAKKKREEEERDRRLGP.

### Genome-wide frameshifting analysis

Chordate mRNA sequences were downloaded from the NCBI RefSeq database in November 2017 and compiled into a BLAST nucleotide database. For a given *H. sapiens* gene name, the human RefSeq transcript with the longest protein-coding CDS out of those with an accession number beginning with “NM_” (i.e., manually curated mRNA sequences) was chosen as the reference sequence; if all accession numbers for the gene began with “XM_” (i.e., computationally annotated mRNA sequences), then one of these with the longest protein-coding CDS was used instead. Reference genes where the longest CDS was <100 codons or where the length of the annotated CDS was not a multiple of 3 were discarded. For each *H. sapiens* reference mRNA sequence, the protein sequence was derived from the translated CDS, and tBLASTn was used to query the protein sequence against the BLAST nucleotide database using the parameter –qcov_hsp_perc 95 to ensure 95% coverage of the query sequence and an *e* value threshold of 0.001. Hits were filtered for ≥65% amino acid identity to the query sequence, and, where multiple sequences from the same taxon were identified, only the hit with highest amino acid identity per taxon was kept. Reciprocal tBLASTn searches (with the same options as above) were performed using each hit from the first search as query sequence and the set of reference mRNAs as the subject sequences and only best reciprocal blast matches were retained. For each gene, the CDS sequences were extracted from each of the identified mRNA sequences and translated, and the amino acid sequences were aligned with MUSCLE (*50*). Using tranalign from EMBOSS, each amino acid alignment was used to guide a corresponding nucleotide alignment. Because of the 65% amino acid identity threshold, these alignments are often restricted to mammalian sequences, but for highly conserved proteins, they can also include sequences from other chordates. In total, alignments were generated for 17,922 human genes, contained a total of 1,586,159 sequences of which 75.1% were mammalian.

We identified all in-frame instances of YCC_UUU_CG in the 17,922 *H. sapiens* CDSs and then counted the number of sequences in the alignment, and the number containing a YCC_UUU_CG aligning exactly to the human YCC_UUU_CG. Of the 134 YCC_UUU_CG matches in human, only 7 were conserved in ≥95% of the aligned sequences. Five of these alignments were phylogenetically restricted (largely primate-specific), containing only 7 to 23 sequences. The remaining two were *ASXL1* (99 sequences, 98 with aligned YCC_UUU_CG) and *PLEKHM2* (114 sequences, 112 with aligned YCC_UUU_CG). Note that the procedure is not exhaustive: There may be instances of YCC_UUU_CG that are conserved in shorter genes (<100 codons) or otherwise missed from the initial set of human reference sequences, conserved in some but not all of the sequences in an alignment (e.g. primate-specific), or conserved in presence but not in precise position across the MUSCLE sequence alignment, and these examples would have been missed by this analysis.

### Cell culture and transfections

For Fig. 3, HEK293T cells (American Type Culture Collection) were maintained in Dulbecco’s modified Eagle’s medium (DMEM) supplemented with 10% fetal bovine serum (FBS), 1 mM l-glutamine, and antibiotics. For Western blotting, cells were transfected with Lipofectamine 2000 reagent (Invitrogen) in six-well plates using the 1-day protocol in which suspended cells are added directly to the DNA complexes in six-well plates, with 1 mg of each indicated plasmid. The transfecting DNA complexes in each well were incubated with 5 × 10^5^ cells suspended in 3 ml of DMEM + 10% FBS and incubated for 36 hours at 37°C in 5% CO_2_.

For GFP-Trap transfections, 1 × 10^7^ cells were forward-transfected with Lipofectamine 2000 reagent in 15-cm petri dishes with 10 mg of each indicated plasmid. Cells were incubated for 48 hours at 37°C in 5% CO_2_.

For dual luciferase assays, cells were transfected with Lipofectamine 2000 reagent, again using the 1-day protocol described above. The following were added to each well: 25 ng of each plasmid plus 0.2 μl of Lipofectamine 2000 in 25 μl of Opti-Mem (Gibco). The transfecting DNA complexes in each well were incubated with 3 × 10^4^ cells suspended in 50 μl of DMEM + 10% FBS at 37°C in 5% CO_2_ for 20 hours.

For polyamine experiments, cells were transfected using Lipofectamine 2000 reagent as described above. Suspended cells were supplemented to a final concentration of 1 mM aminoguanidine hydrochloride (Sigma-Aldrich) or 1 mM aminoguanidine hydrochloride plus 2 mM spermidine (Sigma-Aldrich) before adding to transfecting DNA complexes. Luciferase activities were measured 20 hours after spermidine treatment.

For Fig. 4, HeLa and HEK293T cells were cultured in DMEM (Quality Biological, #112-319-101) supplemented with 2 mM l-glutamine (Gibco, #25030081), 10% fetal bovine serum (BSA) (Corning, #35-011-CV), and penicillin-streptomycin (100 U/ml; Gibco, #15140122) (complete DMEM) in a 37°C incubator (5% CO_2_, 95% air). HeLa cells grown on six-well plates were transiently transfected with 2 μg of plasmid DNA using 8 μl of Lipofectamine 3000 (Invitrogen, #L3000001), according to the manufacturer’s instructions. Approximately 24 hours after transfection, 40,000 cells were replated onto 12-mm coverslips coated with collagen (BioTechne, #3442-050-01). The remaining unseeded cells were used for SDS-PAGE and immunoblotting. Cells were then cultured for an additional 24 hours before fixation and immunofluorescent labeling. For co-immunoprecipitation experiments, HEK293T cells grown on six-well plates were transiently transfected for 48 hours with 1.5 μg of each plasmid DNA and 8 μl of Lipofectamine 3000 (Invitrogen, #L3000001) according to the manufacturer’s instructions.

### Expression constructs

*PLEKHM2* fused dual luciferase constructs (for immunoblotting) were generated by either one-step or two-step polymerase chain reaction (PCR) on *PLEKHM2* gBlock1 Integrated DNA Technologies (IDT) using primer sequences, which incorporated 5′ Xho I and 3′ Bgl II restriction sites. PCR amplicons were digested with Xho I/Bgl II and cloned into Psp XI/Bgl II–digested pDlucV2.0. pDlucV2.0 is a version of pDluc generated by introducing silent mutations into the *Renilla* coding sequence to disrupt two potential donor splice sites (TGGgtaagt).

PLEKHM2-X-GFP and PLEKHM2-K-X-GFP were synthesized as gBlocks with flanking 5′ Sac I and 3′ Bam HI restriction sites and cloned into pcDNA3.4.

*PLEKHM2* unfused dual luciferase constructs (for dual luciferase assay) were generated by either one-step or two-step PCR on a *PLEKHM2* G Block (IDT) using primer sequences, which incorporated 5′ Xho I and 3′ Bgl II restriction sites. PCR amplicons were digested with XhoI/Bgl II and cloned into Psp XI/Bgl II–digested pSGDlucV3.0 (Addgene 119760). *OAZ1* dual luciferase expression constructs were described previously (*52*). All clones were verified by Sanger sequencing (Eurofins). All constructs used for cell biology assays were directly synthesized by Twist Biosciences for overexpression in mammalian cells.

### Antibodies and chemicals

The following primary antibodies were used for immunoblotting (IB) and/or immunofluorescence microscopy (IF) (catalog numbers, sources, and working dilutions are in parentheses): rat anti-HA (12158167001, Roche; IF, 1:500; IB, 1:1,000), mouse anti-LAMP1 (H4A3, DSHB; IF 1:500), rabbit anti-LAMTOR4 (13140, Cell Signaling Technology; IF, 1:500), rabbit anti–myc-tag (2272, Cell Signaling Technology; IB, 1:1000), mouse anti–tubulin–horseradish peroxidase (HRP) (sc-32293, Santa Cruz Biotechnology; IF, 1:1000). Secondary antibodies were as follows: HRP-conjugated goat anti-rat immunoglobulin G (IgG) (H+L) (112-035-003, Jackson Immuno Research; IB, 1:5000), HRP-conjugated goat anti-rabbit IgG (H+L) (111-035-003, Jackson Immuno Research; IB, 1:5000), Alexa Fluor 555–conjugated donkey anti-mouse IgG (A-31570, Thermo Fisher Scientific; IF, 1:1000), Alexa Fluor 555–conjugated goat anti-rabbit IgG (A-21428, Thermo Fisher Scientific; IF, 1:1000), and Alexa Fluor 488–conjugated goat anti-rat IgG (A-11006, Thermo Scientific; IF, 1:1000). Cell edges were outlined by staining of actin with Alexa FluorTM 647–conjugated phalloidin (A22287, Thermo Fisher Scientific; IF, 1:500).

### Dual luciferase assay

Relative light units were measured on a Veritas Microplate Luminometer with two injectors (Turner Biosystems). Transfected cells were lysed in 15 μl of 1 × passive lysis buffer (PLB; Promega), and light emission was measured following injection of 50 μl of either *Renilla* or firefly luciferase substrate. Recoding efficiencies were determined by calculating relative luciferase activities (firefly/*Renilla*) from test constructs and dividing by relative luciferase activities from replicate wells of IFC constructs. Four replicate biological samples were assayed each with four technical repeats. Statistical significance was determined using a two-tailed, homoscedastic Student’s *t* test.

### SDS-PAGE and immunoblotting

For Fig. 3, transfected cells were lysed in 100 ml of 1× PLB. Proteins were resolved by SDS-PAGE and transferred to nitrocellulose membranes (Protran), which were incubated at 4°C overnight with primary antibodies. Immunoreactive bands were detected on membranes after incubation with appropriate fluorescently labeled secondary antibodies using a LI-COR Odyssey Infrared Imaging Scanner.

For Fig. 4, cells were washed twice with ice-cold phosphate-buffered saline (PBS; Corning, #21-040-CM), scraped from the plates in 1x Laemmli sample buffer (1x LSB) (Bio-Rad, #1610747) supplemented with 2.5% v/v 2-mercaptoethanol (Sigma-Aldrich, #60-24-2), heated at 95°C for 5 min, and resolved by SDS-PAGE. Gels were blotted onto nitrocellulose membrane and blocked with 5% (w/v) nonfat milk in tris-buffered saline, 0.1% (v/v) Tween 20 (TBS-T) for 20 min. Membranes were sequentially incubated with primary antibody and secondary HRP-conjugated antibody diluted in TBS-T. SuperSignal West Dura Reagents (Thermo Fisher Scientific, #34075) were used for detection of the antibody signal with a Bio-Rad Chemidoc MP imaging system. β-Tubulin was used as a control for sample loading.

For fig. S5 (I and J), equal amounts of protein were loaded (normalized by a BCA assay) and run on a SDS-PAGE gel. Protein was then transferred to a polyvinylidene difluoride membrane that was blocked with 5% skim milk for 1 hour at 37°C before an overnight incubation with primary antibodies at 4°C. The next day, secondary antibodies were incubated at room temperature for 1 hour before enhanced chemiluminescence measurement. The signal intensity analysis was performed in ImageJ (*53*).

### Immunoprecipitation

For Fig. 3, Cells were lysed in 700 ml of PLB and then incubated with 20 ml of protein G Agarose beads plus anti-HA (3 mg) overnight at 4°C with gentle rocking. The beads were washed with ice-cold 1 × PLB buffer and then removed from the beads by boiling for 5 min in 2× SDS-PAGE sample buffer for SDS-PAGE and Western blotting. For GFP immunoprecipitation, GFP-Trap (Chromtek) was used following the manufacturer’s instructions. Briefly, cells were lysed in 100 ml of NP-40 lysis buffer [10 mM tris/Cl (pH 7.5), 150 mM NaCl, 0.5 mM EDTA, and 0.5% NP-40], and then 95 ml of lysate was diluted to 700 ml in dilution buffer [10 mM tris/Cl (pH 7.5), 150 mM NaCl, and 0.5 mM EDTA] before incubation with 20 ml of GFP-Trap beads for 1 hour at 4°C with gentle rocking. The beads were washed with ice-cold dilution buffer and then removed from the beads by boiling for 5 min in 2× SDS-PAGE sample buffer for SDS-PAGE and Coomassie staining.

For Fig. 4, transfected HEK293T cells were lifted in ice-cold PBS and centrifuged at 500*g* for 5 min. The pellet was washed twice with ice-cold PBS and lysed in 10 mM tris-HCl (pH 7.5), 150 mM NaCl, 0.5 mM EDTA, 0.5% (v/v) NP-40 supplemented with a protease inhibitor cocktail (Roche). Cell lysates were clarified by centrifugation at 17,000*g* for 10 min, and the supernatant (10% was saved as input) was incubated with anti-myc magnetic agarose beads (Thermo Fisher Scientific, #88842) at 4°C for 2 hours. After three washes with 10 mM tris-HCl (pH 7.5), 150 mM NaCl, and 0.5 mM EDTA, the immunoprecipitates were eluted with 1× LSB at 95°C for 5 min. The immunoprecipitated samples and inputs were analyzed by SDS-PAGE and immunoblotting.

For fig. S8, transfected HEK293T cells were lifted in ice-cold PBS and centrifuged at 500*g* for 5 min. The pellet was washed twice with ice-cold PBS and lysed in 10 mM tris-HCl (pH 7.5), 150 mM NaCl, 0.5 mM EDTA, and 0.5% (v/v) NP-40 supplemented with a protease inhibitor cocktail (Roche). Cell lysates were clarified by centrifugation at 17,000*g* for 10 min, and the supernatant (10% was saved as input) was incubated with anti-myc or anti-HA magnetic agarose beads (Thermo Fisher Scientific, #88842) at 4°C for 2 hours. After three washes with 10 mM tris-HCl (pH 7.5), 150 mM NaCl, and 0.5 mM EDTA, the immunoprecipitates were eluted with 1× LSB at 95°C for 5 min. The immunoprecipitated samples and inputs were analyzed by SDS-PAGE and immunoblotting.

### Immunofluorescence microscopy

Cells were seeded on collagen-coated coverslips in 24-well plates at 40,000 cells per well in regular culture medium. After 24 hours, cells were fixed in 4% (w/v) paraformaldehyde (Electron Microscopy Sciences, #15700) in PBS for 20 min, permeabilized and blocked with 0.1% (w/v) saponin, 1% (w/v) BSA (Gold Bio, #A-421-10) in PBS for 20 min, and sequentially incubated with primary and secondary antibodies diluted in 0.1% (w/v) saponin and 1% (w/v) BSA in PBS for 30 min at 37°C. Coverslips were washed three times in PBS and mounted on glass slides using Fluoromount-G (Electron Microscopy Sciences, #17984-24) with 4′,6-diamidino-2-phenylindole (DAPI). Z-stack cell images were acquired on a Zeiss LSM 900 inverted confocal microscope (Carl Zeiss) using a Plan-Apochromat 63X objective (numerical aperture = 1.4) with or without Airyscan detection. Maximum intensity projections were generated with Zeiss ZEN Black software, and final composite images were created using ImageJ (*53*).

### Ribosome profiling

Gencode v.46 transcript ENST00000375799 (4134 base pairs) was used as a representative mRNA of PLEKHM2. The frameshift cassette (UCC_UUU_CGG) is located between the coordinates 1545 and 1553, and frameshifted ribosomes terminate at an early stop codon in the new reading frame (+1) at coordinates 1789 to 1791 (*TAG*). Intuitively, PRF is expected to reduce the number of ribosome-protected fragments aligning downstream of the frameshift-introduced stop codon, and hence, the reduction in read density reflects the efficiency of PRF.This relationship can be mathematically represented as the ratio between read density downstream over the one upstream the frameshift cassette.

To limit the effect of aberrant pauses around translation initiation and termination sites, the 100-nt downstream and upstream, respectively, have been discarded. In a similar reasoning, the region from 50-nt upstream the frameshift cassette and up to 50-nt downstream the frameshift-introduced stop codon has been excluded from the analysis. Specifically, the region between coordinates 340 and 1494 was used to estimate the density of RFPs upstream the frameshift cassette, while coordinates 1842 to 3199 identify the region used to estimate the density downstream of the frameshift-introduced stop codon. TPM (transcripts per kilobase million) values for corresponding regions were obtained from RiboCrypt (https://ribocrypt.org/) for each individual dataset, and to calculate the ratio, the mean value of TPM for each region was considered. The ratios were then log 2–transformed for the further steps of the analysis.

The classification by tissue of origin was based on the curated metadata available in RiboSeq Data Portal (https://rdp.ucc.ie). To assess the statistical significance of the ratio variation, we used a sliding window *z*-score approach similar to what was described earlier (*54*). Briefly, the log 2 ratios from each study were sorted by ascending values of “total counts”—that is to say, the sum of all the raw counts mapping to each nucleotide position in the regions before and after the frameshift cassette. Then, the ratios were grouped into bins of size 300, and descriptive statistics (mean and SD) were calculated and stored. The window was then shifted with a step of 10, and the statistics were recomputed until the end of the available ratios. At that point, each ratio had a collection of means and SDs, which were averaged and used to obtain the *z*-score value for that specific log 2 ratio.

To represent thresholds of *z* scores of 3 and 4 over the log_2 ratio distributions, we used the binning-and-sliding window approach mentioned before to calculate the SDs and means for each value, but with bin size of 50 and step of 10. The intervals between 3 and 4 SDs from the mean were then computed for each datapoint and plotted on the graph as two threshold lines (fig. S3).

### iPSC cell culture, differentiation, purification, and inoculation

iPSCs were generated from the ZZUNEUi022-A cell line (*55*), which was established from male urine cells. PLEKHM2-KO hiSPECs were generated with a single guide RNA targeting the second exon of *PLEKHM2* and verified by sequencing and Western blot^48^. Matrigel-coated six-well plates were seeded with iPSCs at 10,000 cells per well and cultured in PSCeasy medium at 5% carbon dioxide at 37°C. hiSPCs were differentiated into CMs (hiPSC-CMs) using a small molecule–based approach described previously (*42*). On the 30th day of differentiation, lentivirus is added to the hiPSC-CMs at a multiplicity of infection of 4. After a week postinfection, cells were assayed.

### Contractility measurements

Dissociated CMs were reseed onto micropatterned hydrogels with stiffness of 10 kPa. These single cell patterns were arranged in a grid of 1 mm by 1 mm, spaced to minimize the risk of mechanical interaction between adjacent cells. Single-cell contraction force was assessed from single cells using Zeiss fluorescence microscope. We acquired two types of videos via microscopy. Videos of CM contractions were then collected in fluorescence channels, capturing cardiac contractions at 30 frames per second with a 40× objective for 10 s. Cells were imaged in bright field to determine individual cells and beating rates. The video of the movement of fluorescent beads in the substrate caused by the contraction of CMs through the fluorescence channel. Raw video was extracted using Hcell (*56*) software. Briefly, the contraction force was calculated from video of the movement of fluorescent beads in the substrate, and we calculated the magnitude of force vectors (F) from traction stresses. The velocity and contractile force corresponding to each time point are calculated from each frame of the two videos, and the corresponding velocity and contractile force curves are drawn on the basis of these data.

### qPCR of ANP and BNP

mRNA was isolated from the hiSPC-CMs using TRIzol and reverse-transcribed into cDNA with PrimeScript RT Master Mix. Quantitative real-time PCR was performed on samples using a QuantStudio 3 instrument along with TB Green Premix Ex Taq II PCR mix. Glyceraldehyde-3-phosphate dehydrogenase (GAPDH) was used as a housekeeping gene to calculate the relative gene expression using the double delta Cycle Threshold method. The primer sequences used for GAPDH were: GGAGCGAGATCCCTCCAAAAT and GGCTGTTGTCATACTTCTCATGG; for ANP (NPPA): ACAATGCCGTGTCCAACGCAGA and CTTCATTCGGCTCACTGAGCAC; and for BNP (NPPB): TCTGGCTGCTTTGGGAGGAAGA and CCTTGTGGAATCAGAAGCAGGTG

### Calcium transient measurements

Calcium handling was measured as previously described (*42*). Briefly, the green fluorescent calcium–modulated protein 6 fast type (GCaMP6f) calcium imaging system was used to track the calcium transients in hiPSC-CMs. Using the Plexithermo system, spontaneous calcium transients of individual cells on an OLYMPUS IX73 microscope at 37°C were measured, and the data were analyzed using ImageJ (*53*).
