# Supplementary material for: Programmed ribosomal frameshifting during PLEKHM2 mRNA decoding generates a constitutively active proteoform that supports myocardial function
Source: Sci Adv. 2025 Oct 24;11(43):eady1742. doi: 10.1126/sciadv.ady1742 (PMC12551717; doi:10.1126/sciadv.ady1742)
Supplement: Supplementary file 1 — Figs. S1 to S9 [file sciadv.ady1742_sm.pdf]

Science Advances
AAAS

Supplementary Materials for

Programmed ribosomal frameshifting during *PLEKHM2* mRNA decoding generates a constitutively active proteoform that supports myocardial function

Gary Loughran *et al.*

Corresponding author: Yousuf A. Khan, yousuf@stanford.edu; Gary Loughran, g.loughran@ucc.ie; Juan S. Bonifacino, bonifacinoj@mail.nih.gov; Xiaowei Li, fcclixw@zzu.edu.cn

Sci. Adv. **11**, eady1742 (2025)
DOI: 10.1126/sciadv.ady1742

This PDF file includes:

Figs. S1 to S9

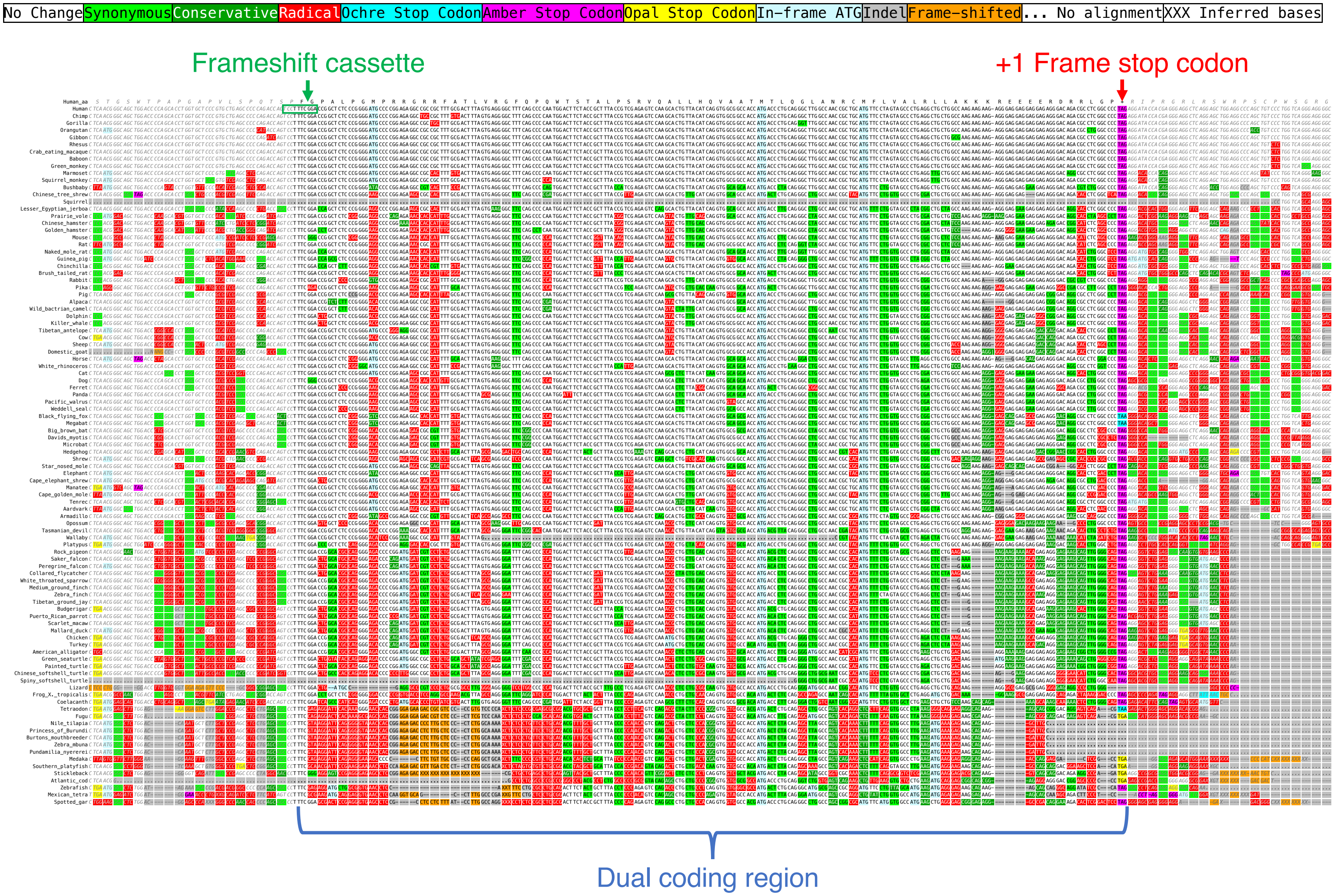

No Change
Synonymous
Conservative
Radical
Ochre Stop Codon
Amber Stop Codon
Opal Stop Codon
In-frame ATG
Indel
Frame-shifted
... No alignment
XXX Inferred bases
Frameshift cassette
+1 Frame stop codon
Dual coding region

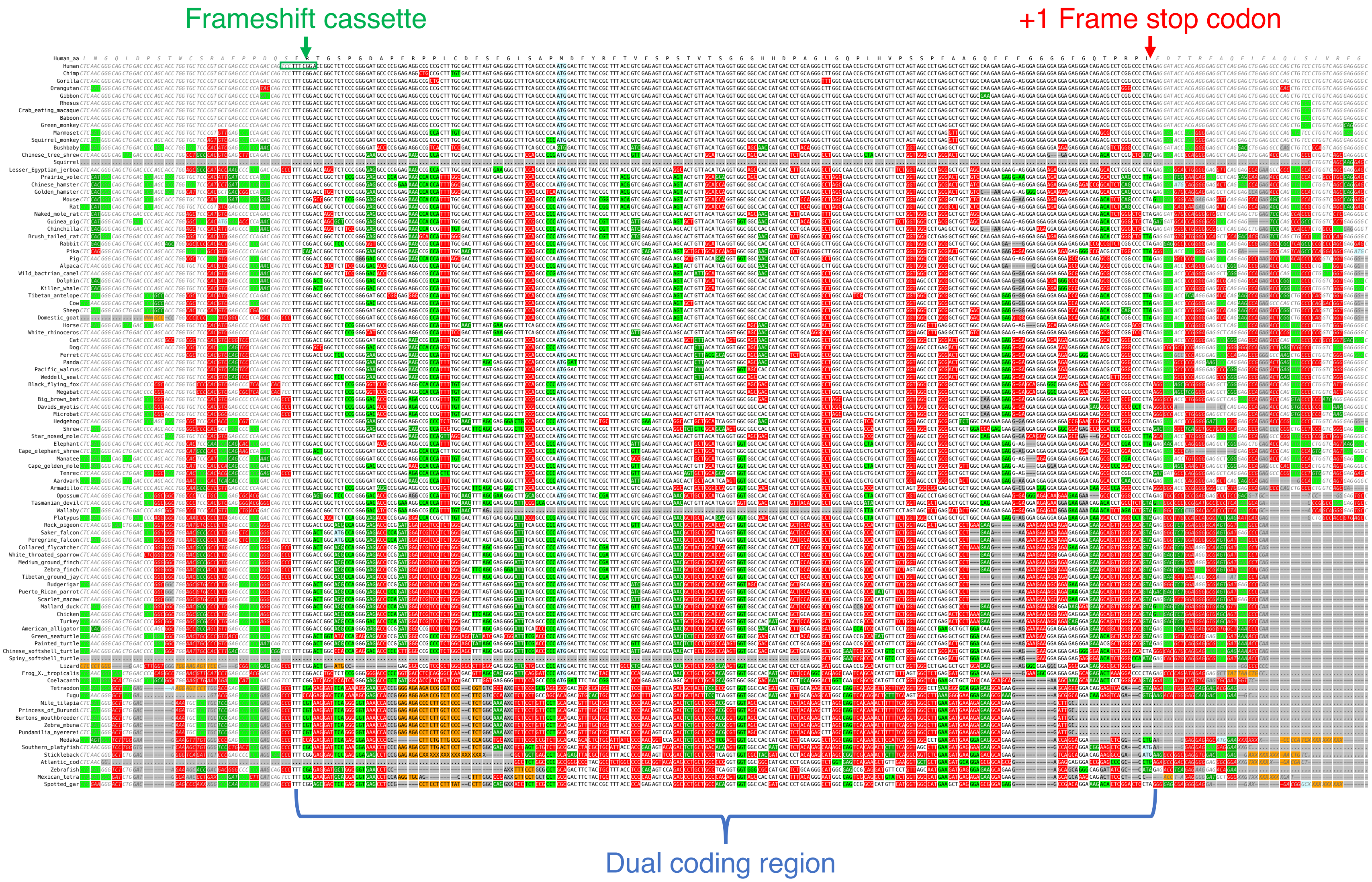
Frameshift cassette
+1 Frame stop codon
Dual coding region

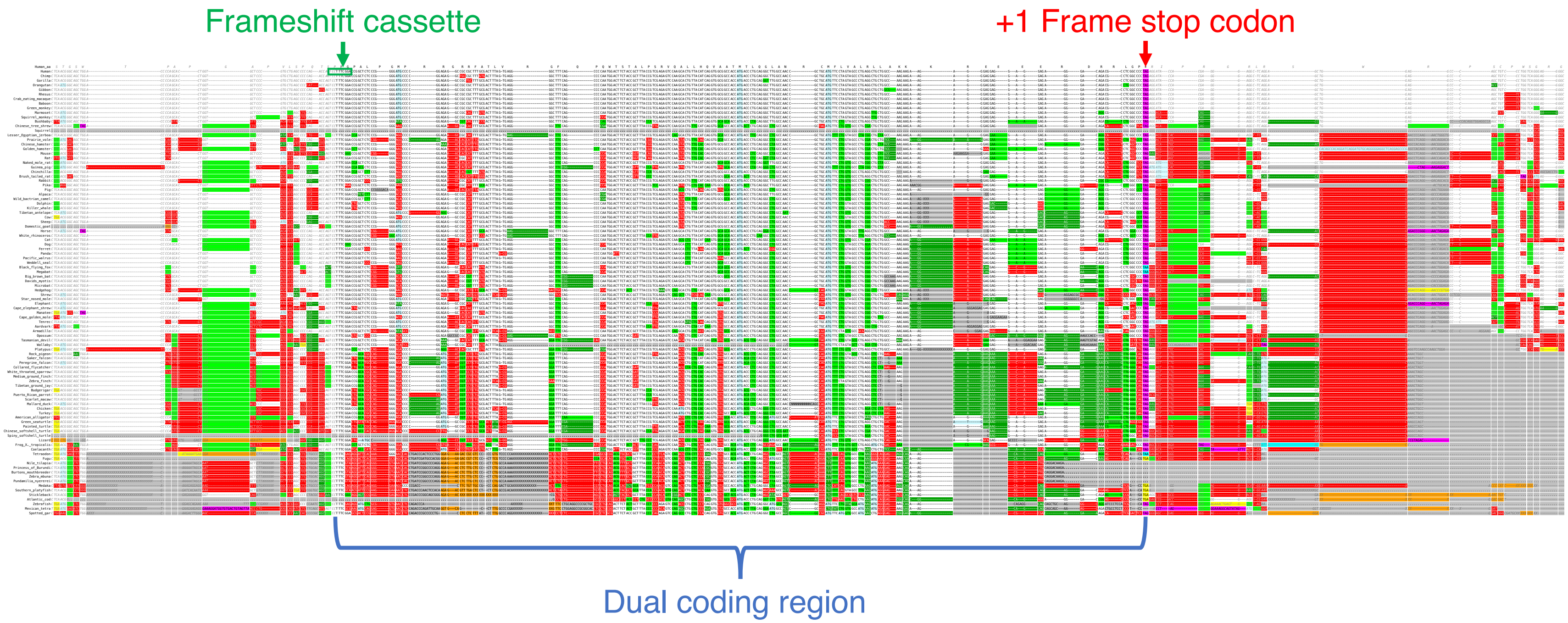
Frameshift cassette
+1 Frame stop codon
Dual coding region

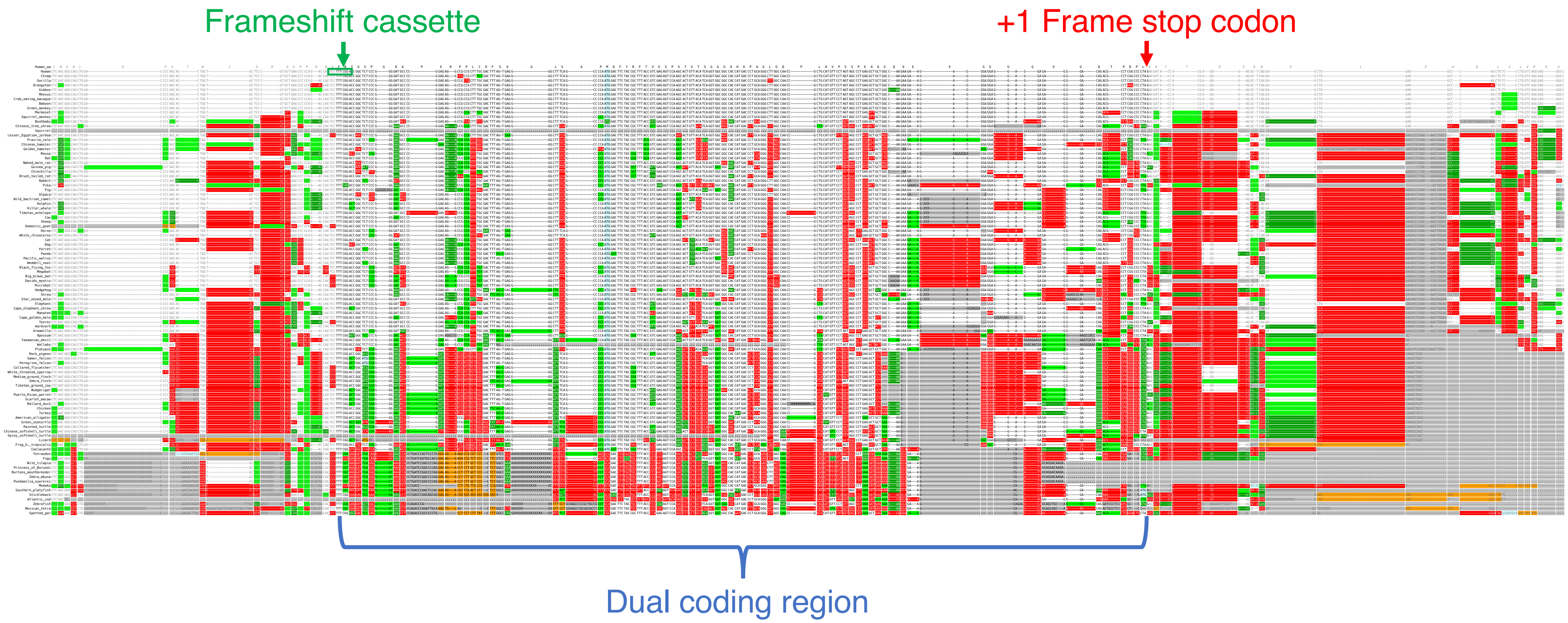
Frameshift cassette
+1 Frame stop codon
Dual coding region

Fig. S1. Genomic alignment of 100 vertebrate species in a neighborhood of the dual coding region, with features relevant to protein-coding evolution color-coded by CodAlignView. For clarity, insertions relative to the human sequence are not shown in (a) and (b); the same alignments including these insertions are shown in (c) and (d), respectively. (a) Codonization and color coding with respect to the +1 frame. Preponderance of synonymous substitutions (light green) in the latter portion of the dual coding region indicates purifying selection on the amino acid sequence translated from this frame. There are many TGA (yellow), TAG (magenta), and TAA (cyan) stop codons in other species in this reading frame both 5' of the PRF cassette (green arrow) and 3' of the +1 frame stop codon (red arrow), but none within the dual coding region. The CCTTTC in the PRF cassette is perfectly conserved in all aligned species. (b) Same region codonized and color-coded with respect to the zero frame.

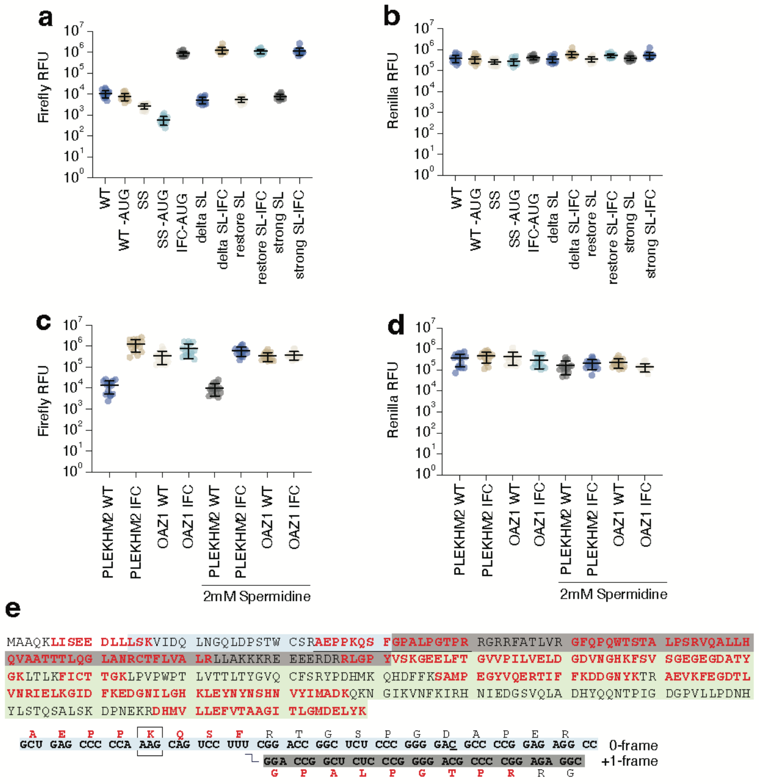

Fig. S2. PLEKHM2 luciferase assays and additional mass-spectrometry data. **a.** Absolute firefly luciferase values. **b.** Absolute *Renilla* luciferase values; all values are within the same order of magnitude. **c.** Absolute firefly luciferase values in spermidine experiments. **d.** Absolute *Renilla* luciferase values in spermidine experiments. **e.** Mass-spectrometry results of PLEKHM2-K-GFP constructs; covered peptides are shown in red font with the junction peptide underlined. The transition of the reading frame is shown below.

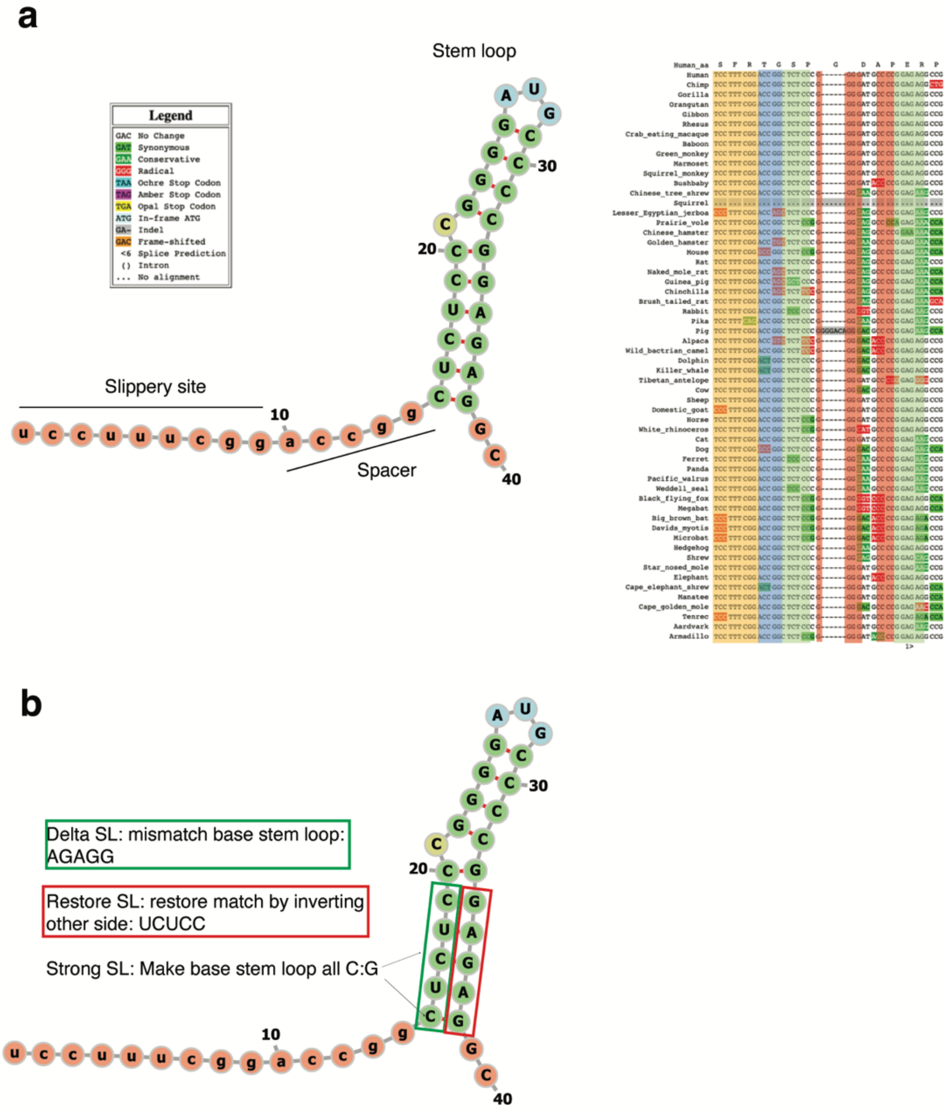

Fig. S3. Potential RNA secondary structure downstream of slippery site. **a.** Predicted RNA secondary structure using forna[56] and CodAlignView with secondary structure regions highlighted. Uppercase letters in the diagram indicate which nucleotides were inputted for structure prediction. **b.** Diagram showing what structural mutants were made.

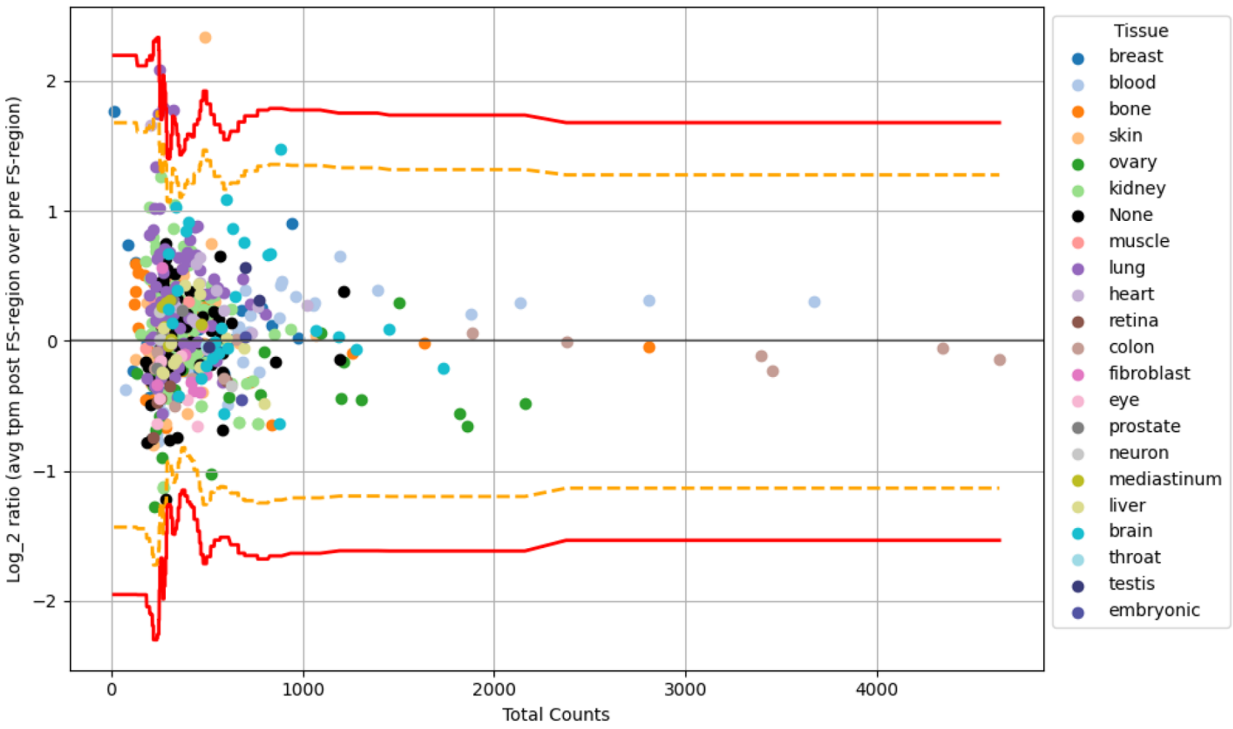

Fig. S4. Ribosome profiling analysis mined from across different tissues. Individual datasets are represented by dots coloured by the tissue of origin. The orange dashed line and red solid line represent three and four z-score thresholds, respectively. The y-axis shows the log2 ratio of ribosome protected fragments mapped downstream of the +1 frame stop codon over upstream (see Methods). The x-axis shows the total number of fragments aligned to the considered region of the PLEKHM2 mRNA.

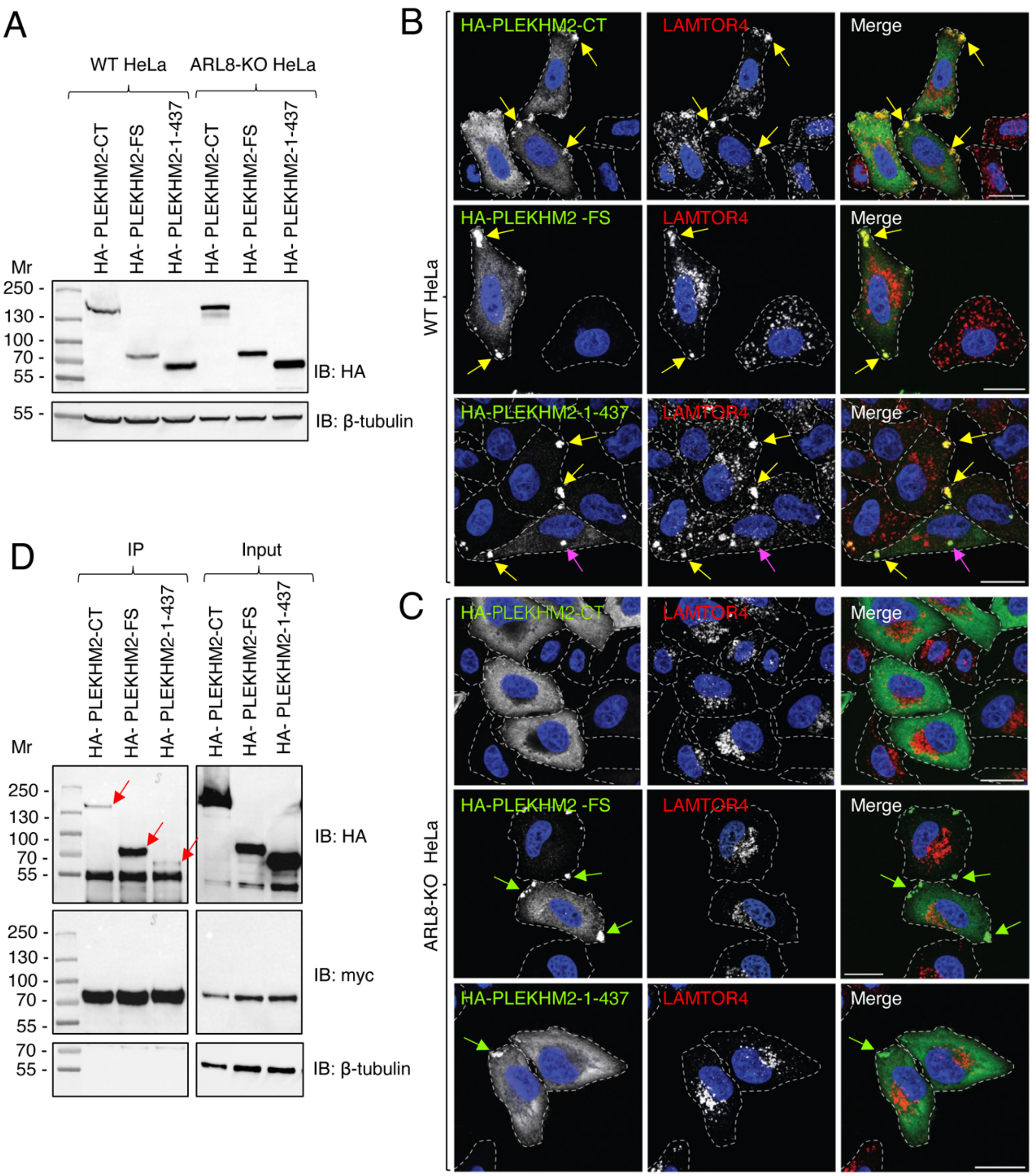

Fig. S5. Expression, activity, and self-association of PLEKHM2-FS. a. WT or ARL8-KO HeLa cells were transiently transfected with the indicated HA-tagged PLEKHM2 constructs (see scheme in Fig. 4a) for 48 h and analyzed by SDS-PAGE and immunoblot analysis with antibodies to the HA epitope. β-tubulin was used as a loading control. The positions of molecular mass markers (Mr, in kDa) are indicated on the left. **b.** WT HeLa cells like those shown in panel **a** were analyzed by confocal immunofluorescence microscopy for the HA epitope (green) and

endogenous LAMTOR4 (lysosomes, red). Nuclei were labeled with DAPI (blue). Cell edges were outlined by staining of actin with fluorescent phalloidin (not shown) and indicated by dashed lines. Arrows indicate clusters of HA-PLEKHM2 together with lysosomes (yellow arrows) or without (green arrows) at the cell tips or more centrally (magenta arrows). Scale bars: 20 µm. Notice how all HA-PLEKHM2 constructs drive distribution of both HA-PLEKHM2 and lysosomes to cell tips. **c.** ARL8-KO HeLa cells were transfected and analyzed as described for panel **b**. Scale bars: 20 µm. Notice that HA-PLEKHM2-CT is unable to localize to and drive lysosomes to cell tips in ARL8-KO cells. In contrast, HA-PLEKHM2-FS can localize to, but cannot drive lysosomes to cell tips. HA-PLEKHM2-1-437 is incapable of moving either protein to cell tips. **d.** HEK293T cells were co-transfected with plasmids encoding the indicated HA-tagged constructs with myc-PLEKHM2-FS and subjected to immunoprecipitation with an antibody to the myc epitope. Immunoprecipitates (IP) and cell extracts (10%, Input) were analyzed by SDS-PAGE and immunoblotting (IB) for the HA and myc epitopes, or β-tubulin (loading control). Red arrows indicate the HA constructs that are co-immunoprecipitated with the myc-PLEKHM2-FS construct. The positions of molecular mass markers (Mr, in kDa) are indicated on the left. Notice the much greater co-immunoprecipitation of myc-PLEKHM2-FS with HA-PLEKHM2-FS relative to HA-PLEKHM2-CT and HA-PLEKHM2-1-437.

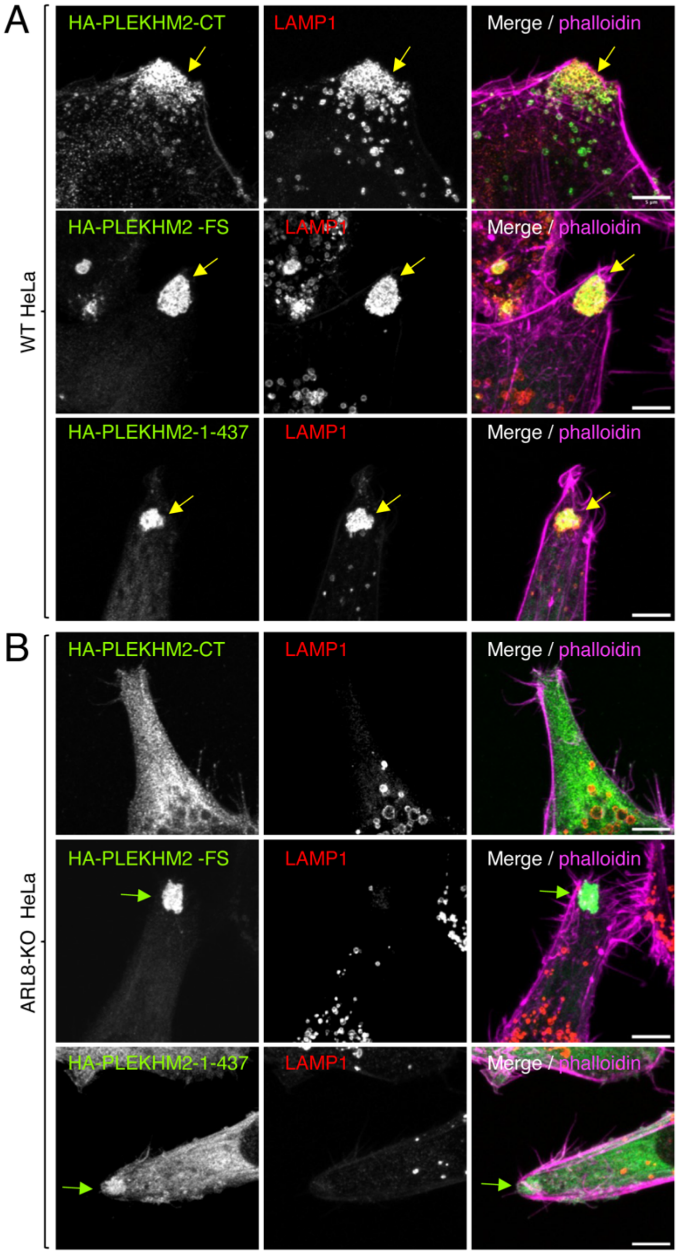

Fig. S6. Airyscan microscopy of WT and ARL8-KO HeLa cells expressing different PLEKHM2 variants. a. WT HeLa cells were transiently transfected with the indicated HA-tagged PLEKHM2 constructs for 48 h and analyzed by immunofluorescence microscopy for the HA epitope (green), endogenous LAMP1 (red) and Alexa Fluor™ 647-conjugated phalloidin (magenta). Cells were imaged using a higher-resolution Airyscan confocal microscope. Arrows indicate clusters of PLEKHM2 together with lysosomes (yellow arrows) or without (green arrows) at cell tips outlined by Alexa Fluor™ 647-conjugated phalloidin. Scale bars: 5 µm.

Notice that HA-PLEKHM2-FS or HA-PLEKHM2-1-437 form tight clusters with lysosomes. **b.** ARL8-KO HeLa cells were transfected, stained, and imaged as described for panel **a**. Notice the absence of HA-PLEKHM2 and the reduction of HA-PLEKHM2-1-437 at cell tips. Scale bars: 5 μm.

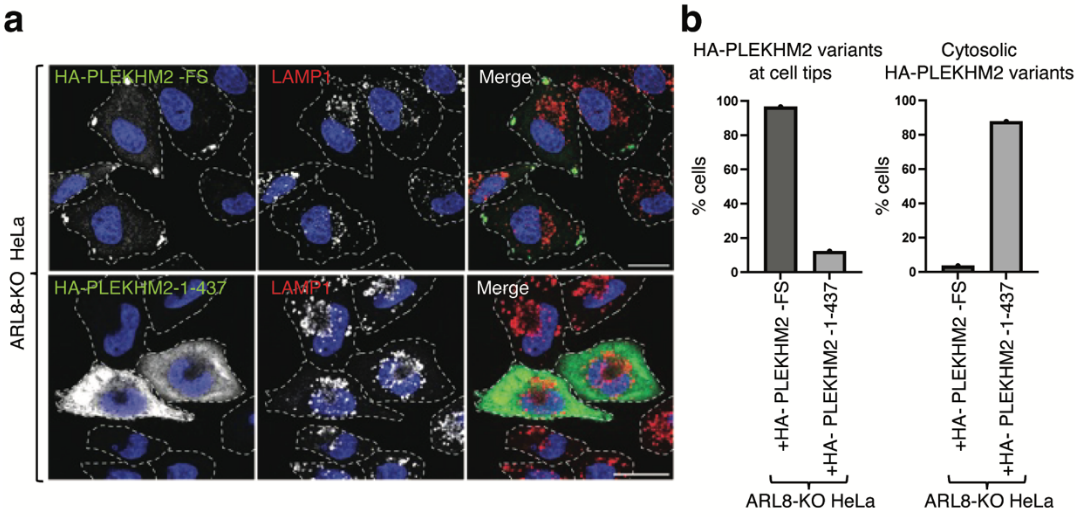

Fig. S7. Enhanced ARL8-independent localization of PLEKHM2-FS to cell tips. a. ARL8-KO HeLa cells were transiently transfected with the indicated HA-tagged PLEKHM2 constructs for 48 h and analyzed for confocal immunofluorescence microscopy for the HA epitope (green), and endogenous LAMP1 (lysosomes, red). Nuclei were labeled with DAPI (blue). Cell edges were outlined by staining of actin with fluorescent phalloidin (not shown) and indicated by dashed lines. Scale bars: 20 µm. **b.** Quantification of the percentage of cells displaying localization of HA-PLEKHM2 variants to cell tips (left panel) or cytosol (right panel) from more than 150 cells per sample. Notice that HA-PLEKHM2-1-437 is less tip-localized and more cytosolic relative to HA-PLEKHM2-FS, suggesting a role for the transframe sequence in ARL8-independent localization of HA-PLEKHM2-FS to cell tips, indicative of interaction with kinesin-1.

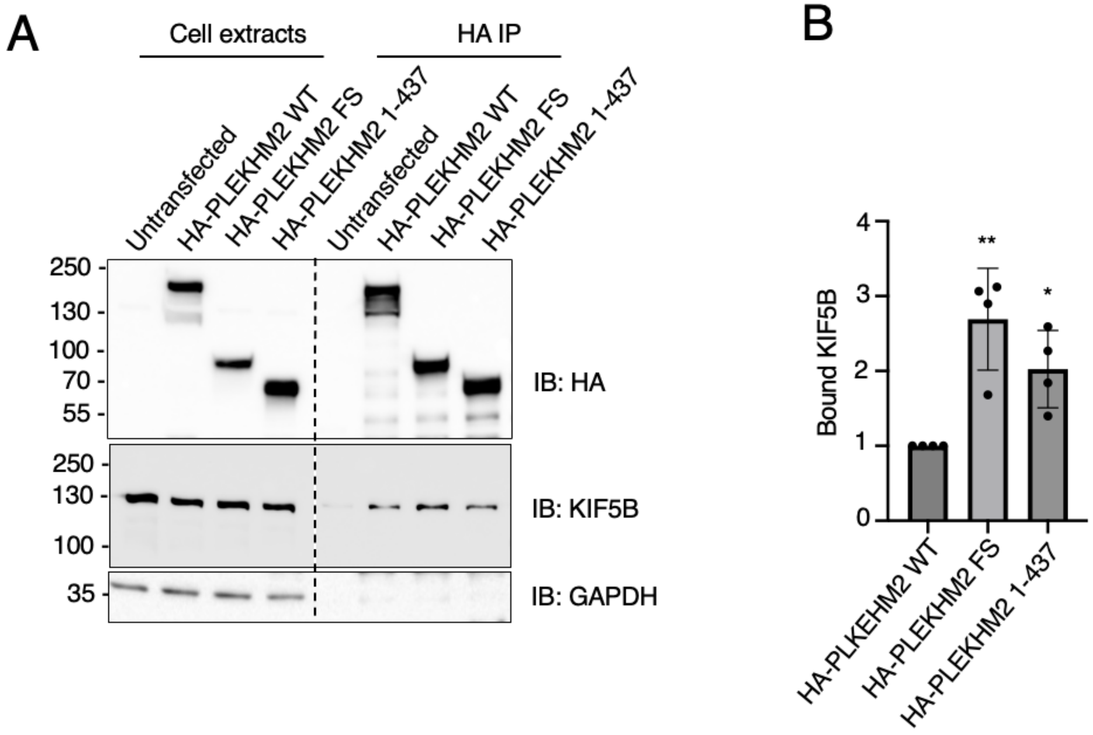

Fig. S8. a. HEK293T cells were transfected with plasmids encoding the indicated HA-tagged constructs and subjected to immunoprecipitation with an antibody to the HA epitope. Untransfected cells were used as control. Immunoprecipitates (HA IP) and cell extracts were analyzed by SDS-PAGE and immunoblotting (IB) for the HA epitope and endogenous KIF5B, or GAPDH (loading control). The positions of molecular mass markers are indicated on the left. Notice the much greater co-immunoprecipitation of endogenous KIF5B with HA-SKIP-FS relative to HA-PLEKHM2 and HA-PLEKHM2-1-437. **b.** Quantification from four independent experiments such as those shown in panel A. Values are the mean ± SD. Statistical significance was calculated by one-way ANOVA followed by multiple comparisons using Dunnett's test. *p< 0.05; **p< 0.01.

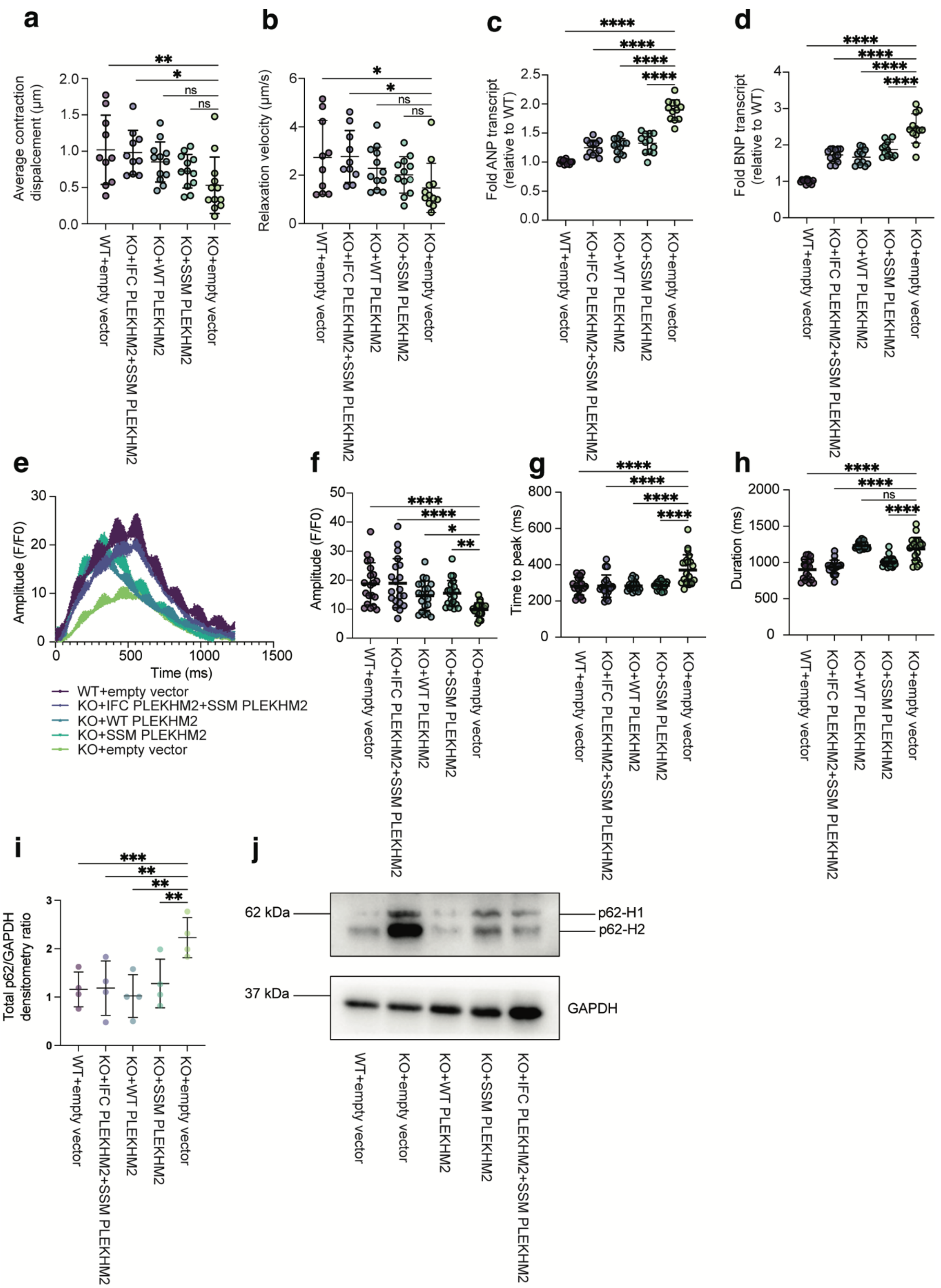

a
b
c
d
e
f
g
h
i
j
Average contraction dispalcement (μm)
Relaxation velocity (μm/s)
Fold ANP transcript (relative to WT)
Fold BNP transcript (relative to WT)
Amplitude (F/F0)
Time (ms)
Time to peak (ms)
Duration (ms)
Total p62/GAPDH densitometry ratio
WT+empty vector
KO+IFC PLEKHM2+SSM PLEKHM2
KO+WT PLEKHM2
KO+SSM PLEKHM2
KO+empty vector
62 kDa
37 kDa
p62-H1
p62-H2
GAPDH

Fig. S9. Additional assays of PLEKHM2-KO hiPSC-CMs and WT hiPSC-CMs. a-b. Quantification of average contraction and relaxation velocity (n = 10-12). c-d. qPCR of ANP and BNP transcripts, with fold ANP (NPPA) or BNP (NPPB) transcript calculated relative to WT (n = 12). e. Representative single trace of calcium transient. f-h. Quantification of amplitude, time to peak, and duration of calcium transients in hiPSC-CMs (n = 20-22). i. Western blot quantifications of p62 isoforms (n=4). j. Representative western blot showing p62 (H1 and H2 isoforms). All statistical tests performed were aone-way ANOVA (ns: p-value > 0.05, *: p-value < 0.05, **: p-value <0.01, ***: p-value <0.001, ****:p-value<0.0001).
